# Comparative Volatile Profiling Reveals the Chemical Association of Huangshui with Fermented Grains, Pit Mud, and Nongxiangxing Baijiu

**DOI:** 10.3390/foods15152722

**Published:** 2026-08-02

**Authors:** Wei Cheng, Na Li, Qingyun Zhu, Gengdian Liu, Xiaoyun Hao, Chao Jiang, Lele Zhang, Xianfeng Du

**Affiliations:** 1 School of Biology and Food Engineering, Fuyang Normal University, Fuyang 236037, China; 18365297578@163.com (N.L.); 17201518161@163.com (Q.Z.); 2 School of Chemical Engineering, Xi’an University, Xi’an 710065, China; gengdiancsy@163.com (G.L.); haoxiaoyun2026@126.com (X.H.); 3 Anhui WenWang Brewery Co., Ltd., Fuyang 236400, China; jiangchao_ww@hengshuilaobaigan.net; 4 School of Food and Nutrition, Anhui Agricultural University, Hefei 230036, China; dxf@ahau.edu.cn

**Keywords:** Nongxiangxing baijiu, Huangshui, fermented grains, pit mud, volatile organic compounds

## Abstract

Huangshui (HS), fermented grains (FG), and pit mud (PM) are closely connected during the fermentation of Nongxiangxing baijiu (NXB); however, the contribution of HS to the differences in flavor profiles across PM, FG, and distilled baijiu has not been fully elucidated. Herein, instrumental techniques of electronic nose (E-nose), headspace gas chromatography–ion mobility spectrometry (HS-GC-IMS), and headspace solid-phase microextraction coupled with gas chromatography–mass spectrometry (HS-SPME-GC-MS) were used to compare the volatile profiles of HS, FG, PM, and two quality grades of NXB. E-nose analysis revealed that PM exhibited stronger signals related to sulfur-containing, organic, and terpene-associated compounds. Gas chromatography indicated that special-grade baijiu had a more favorable ethyl caproate/ethyl lactate ratio than superior-grade baijiu. Furthermore, HS-GC-IMS revealed different volatile profiles of the samples. Up to 183 volatile organic compounds (VOCs) were identified via HS-SPME-GC-MS, with esters and acids being the dominant classes. Multivariate analysis showed close relationships among HS, FG, and PM, and 35 VOCs were identified as important discriminatory compounds. These results suggest the differences and similarities in VOCs among the HS, FG, PM, and NXB samples, indicating that HS is chemically associated with both FG and PM. This study provides useful insights into HS utilization and quality regulation of NXB through the effective management of HS.

## 1. Introduction

Nongxiangxing baijiu (NXB), one of the most representative aroma types of Chinese baijiu, is produced through spontaneous solid-state fermentation in a mud pit. This process involves a complex and dynamic microecosystem in which microorganisms from fermented grains (FGs), pit mud (PM), and the surrounding fermentation environment continuously transform raw materials into various flavor-active compounds [1,2,3]. The aroma quality of NXB is primarily determined by the composition, concentration, and balance of volatile compounds distilled from FG, particularly esters, alcohols, acids, aldehydes, ketones, and other trace aroma compounds [4]. Therefore, understanding the factors that regulate the formation, accumulation, and transformation of flavor compounds in FG is essential for improving the quality and stability of NXB [5,6,7].

During fermentation of baijiu, huangshui (HS) gradually forms and accumulates at the bottom of the fermentation pit. HS is a yellowish acidic liquid rich in water-soluble nutrients, organic acids, alcohols, esters, and other microbial metabolites derived from FG and microbial metabolism [8,9]. Owing to its liquid state and direct contact with FG and PM, HS may facilitate migration, dissolution, and exchange of metabolites among different fermentation niches. Additionally, PM is generally regarded as an important microbial habitat and flavor precursor reservoir in NXB fermentation, whereas FG is a direct substrate for distillation and aroma formation [10,11]. In this context, HS may act as an intermediate metabolic phase related to PM and FG. However, the contribution of HS to the differences in flavor profiles across PM, FG, and distilled baijiu has not been fully elucidated.

In recent years, different instrumental techniques have been widely used to characterize volatile organic compounds (VOCs) in baijiu and its fermentation matrices, including gas chromatography–flame ionization detection (GC-FID), gas chromatography–mass spectrometry (GC-MS), comprehensive two-dimensional gas chromatography–time-of-flight mass spectrometry (GC×GC-TOF-MS), gas chromatography–ion mobility spectrometry (GC-IMS), and electronic nose (E-nose) analysis [12,13,14]. These techniques provide complementary information on the volatile profiles, aroma differences, and characteristic compounds in baijiu fermentation systems. Previous studies have identified several key aroma compounds and investigated the effects of functional microorganisms, fermentation conditions, and raw materials on baijiu quality [3,14,15]. Nevertheless, most studies have focused on baijiu, FG, or PM individually, whereas the similarities and differences in the flavor profiles of HS, FG, PM, and NXB remain poorly understood, and the chemical association of HS with these fermentation matrices has not been systematically investigated [8,16,17]. These knowledge gaps limit our understanding of HS characteristics and their potential relevance to NXB flavor formation and quality regulation.

Therefore, this study aims to characterize the flavor profiles of HS, FG, and PM collected from the bottom of an NXB fermentation pit and to compare these profiles with those of two quality grades of distilled NXB. E-nose, headspace GC-MIS (HS-GC-MIS), and headspace solid-phase microextraction (HS-SPME)-GC-MS were used to comprehensively analyze the VOCs of these samples. We hypothesized that HS acts as an intermediate flavor-associated matrix reflecting the metabolic characteristics of PM and FG and thus participates in the flavor formation of NXB. Furthermore, we analyzed the similarities and differences in flavor profiles between HS and the final distilled NXB. This study provides new insights into the flavor profiles of HS, FG, PM, and NXB samples, offering a reference for HS utilization and quality control of NXB in production.

## 2. Materials and Methods

### 2.1. Sampling and Reagents

#### 2.1.1. Sample Collection and Preparation

FG, HS, and PM samples were collected from the bottom region of an NXB fermentation pit at the end of fermentation. The baijiu samples were obtained by distilling the collected FG. All samples were provided by a baijiu producer located in Linquan City, Anhui Province, China (32.87° N, 115.19° E). The distilled baijiu samples were classified into two quality grades according to the producer’s quality evaluation system: special-grade baijiu, abbreviated as JD, with an alcohol content of 77.0 ± 0.1% vol; and superior-grade baijiu, abbreviated as YZ, with an alcohol content of 72.0 ± 0.1% vol.

After collection, all samples were immediately sealed and transported to the laboratory under refrigerated conditions. Before analysis, FG, HS, and PM samples were stored at 4 °C. For JD and YZ, 450 mL of each baijiu sample was collected and divided into three portions: 200 mL for the determination of total acids and esters, 50 mL for VOC analysis, and 200 mL for E-nose analysis. Because ethanol concentration may influence VOC partitioning during headspace analysis, all baijiu samples were diluted to the same ethanol concentration (10% vol) with ultrapure water before HS-GC-IMS, HS-SPME-GC-MS, and E-nose analyses to minimize ethanol-related matrix effects and improve comparability.

FG, HS, and PM samples were collected at the end of fermentation from three independent NXB fermentation pits operated under the same conditions and during the same fermentation cycle. In each pit, five sampling positions were selected from the bottom region to ensure within-pit spatial heterogeneity. For each matrix, equal amounts of material collected from the five positions were pooled to generate one composite FG, HS, and PM sample per pit. Consequently, the three independent pits served as three biological replicates for each matrix (*n* = 3). The five sampling positions within each pit were considered subsamples and were not treated as independent replicates. Unless otherwise stated, each composite sample was analyzed in triplicate for each instrumental method.

#### 2.1.2. Chemicals and Reagents

A mixed standard solution containing six ketones (2-butanone, 2-pentanone, 2-hexanone, 2-heptanone, 2-octanone, and 2-nonanone) was purchased from Aladdin Industrial Corporation (Shanghai, China). Pentyl acetate (≥98.0%, analytical grade), anhydrous ethanol (≥99.8%, chromatographic grade), and 2-methyl-3-heptanone (≥95.0%, chromatographic grade) were also obtained from Aladdin Industrial Corporation. A mixture of *n-*alkanes (C7–C30) was purchased from Sigma-Aldrich (St. Louis, MO, USA). Sodium chloride (≥98.5%, analytical grade) was purchased from Sinopharm Chemical Reagent Co., Ltd. (Shanghai, China). Ultrapure water was obtained using a Milli-Q water purification system (Millipore, Burlington, MA, USA).

### 2.2. E-Nose Analysis

The overall volatile profiles of FG, HS, PM, JD, and YZ were analyzed using an E-nose system. For solid samples, the FG or PM (5.0 g) was placed in a headspace vial. The vial was sealed and equilibrated at 50 °C with shaking at 40 Hz for 10 min, followed by incubation for 20 min. For liquid samples, 5.0 mL of HS or a diluted baijiu sample was transferred to a headspace vial and sealed for 30 min before analysis. After equilibration, the headspace gas was introduced into the system. The operating parameters were as follows: sampling time, 1 s; zero-adjustment time, 5 s; sample waiting time, 5 s; detection time, 100 s; cleaning time, 100 s; sensor chamber flow rate, 300 mL/min; and sample injection flow rate, 300 mL/min. The sensor response was recorded from 90 s to 92 s when the signals were stable, and the obtained values were averaged for subsequent data analysis. Each sample was analyzed in triplicate. The E-nose operating procedure was based on a previously reported method [18]. The performance characteristics of the E-nose system are listed in Table 1.

### 2.3. Determination of Total Acids, Total Esters, and Major Volatile Compounds in Baijiu

The total acid and ester contents of the JD and YZ samples were determined according to a previously described method [19] based on the Chinese standards for baijiu analysis [20]. The concentrations of the major volatile compounds in baijiu, including ethyl acetate, ethyl caproate, ethyl lactate, 1-propanol, 2-butanol, isobutyl alcohol, 1-butanol, and isoamyl alcohol, were determined via GC-FID following the same standard protocols [20]. The results are expressed as g/L of the baijiu sample.

### 2.4. VOC Detection via HS-GC-IMS and Data Analysis

#### 2.4.1. Sample Pretreatment and HS-GC-IMS Conditions

VOCs in the FG, HS, PM, JD, and YZ samples were analyzed using a FlavourSpec^®^ GC-IMS system (G.A.S., Dortmund, Germany) according to previously reported methods [21], with slight modifications.

For liquid samples, 1.0 mL of HS or diluted baijiu sample was placed in a 20 mL headspace vial. For solid samples, FG or PM (1.0 g) was placed in a 20 mL headspace vial. Then, 10 μL of 2-methyl-3-heptanone internal standard solution was added to each vial. The vials were sealed and incubated at 60 °C for 15 min. The headspace injection volume was 100 μL, and the injection was performed in splitless mode.

The GC-IMS system was equipped with an ITEX dynamic headspace module (CTC Analytics AG, Zwingen, Switzerland) and an MXT-WAX capillary column (15 m × 0.53 mm × 1.0 μm; Restek, Bellefonte, PA, USA). High-purity nitrogen (≥99.999%) was used as the carrier and drift gas. The temperature of the GC column was maintained at 60 °C. The injection needle temperature was set at 130 °C. The adsorption and desorption temperatures were set at 90 and 150 °C, respectively. The de-ethanolization temperature was set at 90 °C, and the ethanol removal time was 60 s. The dynamic headspace extraction was conducted for 12 cycles using Tenax TA as the adsorbent [21]. The carrier gas flow program was set according to Cheng et al. [21]. The parameters are listed in Table 2.

#### 2.4.2. Identification of VOCs by GC-IMS and Data Processing

The retention index (RI) of each compound was calculated using the retention times of mixed ketone standards. VOCs detected by GC-IMS were tentatively identified by matching the retention indices and drift times with entries in the VOCal library. Because authentic standards were unavailable for all detected compounds, the reported identifications should be regarded as putative [12,21,22]. The relative signal intensity of each compound was used for comparative analysis, and semi-quantitative analysis was performed according to previously described methods [21,23].

Three-dimensional spectra, two-dimensional topographic plots, differential spectra, and fingerprint plots were generated using the VOCal software (version 0.16.0, G.A.S., Dortmund, Germany) and its built-in Reporter and Gallery Plot plugins [21,24]. These visualizations were used to compare the VOC differences among the FG, HS, PM, JD, and YZ samples.

### 2.5. VOC Detection via HS-SPME-GC-MS and Data Analysis

#### 2.5.1. VOC Extraction from FG Samples

The VOCs in the FG samples were extracted according to the method reported by Hu et al. [18] with minor modifications. Briefly, 20.0 g of FG was mixed with 40 mL of boiled ultrapure water containing 1.0% anhydrous CaCl_2_. The mixture was soaked overnight at 4 °C, ultrasonicated in an ice-water bath for 30 min, and centrifuged at 10,000 r/min for 20 min at 4 °C. Subsequently, 5.0 mL of the supernatant was transferred into a 20 mL headspace vial containing 3.0 g of NaCl and 20 μL of pentyl acetate internal standard solution at 0.0322 g/L. A DVB/CAR/PDMS fiber (50/30 μm; Supelco, Bellefonte, PA, USA) was used for HS-SPME extraction. The sample was equilibrated at 50 °C for 5 min and extracted for 45 min with stirring at 250 rpm. After extraction, the fibers were thermally desorbed at 250 °C for 5 min at the injection port of the GC-MS system. The GC-MS analysis was performed using an Agilent 8860 GC system coupled to an Agilent 5977 B MSD detector (Agilent Technologies) equipped with a DB-FFAP capillary column (60 m × 0.25 mm × 0.25 μm). The GC-MS operating conditions were set according to Hu et al. [18].

#### 2.5.2. VOC Extraction from HS Samples

The VOCs in the HS samples were extracted as described by Gao et al. [9], with minor modifications. Briefly, 2.0 mL of HS was mixed with 3.0 mL of ultrapure water in a 20 mL headspace vial, followed by the addition of 20 μL of 2-octanol internal standard solution at 0.0424 g/L. The vial was then sealed and equilibrated at 60 °C for 15 min with magnetic stirring. VOCs were extracted using a DVB/CAR/PDMS fiber for 50 min at 60 °C with stirring at 300 rpm. After extraction, the fiber was immediately inserted into the injection port of an Agilent 8860 GC system coupled with an Agilent 5977B MSD detector and desorbed at 250 °C for 5 min. Separation was performed using an HP-INNOWAX capillary column (30.0 m × 0.25 mm × 0.25 μm; Agilent Technologies, Santa Clara, CA, USA). The GC-MS operating conditions were set according to Kang et al. [17].

#### 2.5.3. VOC Extraction from PM Samples

The VOCs in the PM samples were extracted according to the method described by Zhang et al. [10], with minor modifications. Briefly, 1.0 g of PM was placed into a 20 mL headspace vial, followed by adding 10 μL of pentyl acetate internal standard solution at 0.0322 g/L. The vial was sealed and equilibrated at 50 °C for 15 min. The VOCs were extracted using a DVB/CAR/PDMS fiber for 45 min while stirring at 250 rpm. After extraction, the fiber was desorbed at 250 °C for 5 min in the injection port of an Agilent 8860 GC system coupled with an Agilent 5977B MSD detector. Separation was performed using a DB-FFAP capillary column (60 m × 0.25 mm × 0.25 μm; Agilent Technologies, Santa Clara, CA, USA). The GC-MS operating conditions were set according to Zhang et al. [10].

#### 2.5.4. VOC Extraction from Baijiu Samples

Before HS-SPME-GC-MS analysis, the JD and YZ samples were diluted to 10.0% vol with deionized water. Then, 5.0 mL of each diluted baijiu sample was transferred into a 20 mL headspace vial containing 1.8 g of NaCl. Subsequently, 20 μL of pentyl acetate internal standard solution at 0.05784 g/L was added, and the vial was immediately sealed. A DVB/CAR/PDMS fiber was used for VOC extraction. The samples were pre-equilibrated at 50 °C for 5 min and then extracted for 45 min. After extraction, the fiber was immediately inserted into the GC injection port and desorbed at 250 °C for 5 min. The GC-MS analysis was performed using an Agilent 8860 GC system coupled with an Agilent 5977B MSD detector equipped with a DB-FFAP capillary column (60 m × 0.25 mm × 0.25 μm). Helium with a purity of ≥99.999% was used as the carrier gas at a flow rate of 1.0 mL/min. The oven temperature and GC-MS conditions were set according to Cheng et al. [14].

#### 2.5.5. Identification and Semi-Quantification of VOCs by GC-MS

The VOCs were identified by comparing their mass spectra with those in the NIST12 mass spectral library. Compounds with a spectral similarity score of >800 and a matching degree of >80% were retained for further analyses. The RI information calculated using C7–C40 *n*-alkanes was also used to assist in compound identification. The relative concentration of each VOC was calculated on the basis of the peak area ratio between the target compound and the corresponding internal standard according to previously described methods [5,14]. For comparative analysis, the detected VOCs were classified into esters, alcohols, acids, aldehydes, ketones, aromatics, phenols, furans, sulfur-containing compounds, terpenes, and acetals.

### 2.6. Statistical Analysis

All quantitative results are expressed as mean ± standard deviation of biological replicates. One-way analysis of variance (ANOVA) was performed using IBM SPSS Statistics (version 26.0) to evaluate differences among sample groups. When ANOVA indicated significant differences, Duncan’s multiple-range test was used for post hoc comparisons. A *p*-value of <0.05 was considered statistically significant, while a *p*-value of <0.01 was considered highly significant. Different lowercase letters indicate significant differences among groups based on Duncan’s test. Principal component analysis (PCA) and partial least squares discriminant analysis (PLS-DA) were performed to visualize overall sample patterns and identify variables contributing to group discrimination. The significance of sample separation in PCA and PLS-DA was not evaluated by ANOVA. Instead, PLS-DA model robustness was assessed using cross-validation and permutation testing.

## 3. Results and Discussion

### 3.1. E-Nose Analysis of FG, HS, PM, JD, and YZ Samples

**Figure 1:**
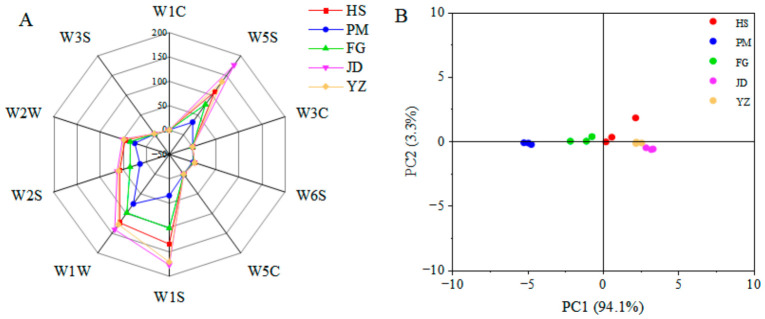
Radar chart of aroma characteristics and PCA score plot of five different samples based on E-nose: (**A**) Radar chart of aroma characteristics. (**B**) PCA score plot of five different samples.

The E-nose sensor responses showed clear differences among FG, HS, PM, JD, and YZ (Figure 1), suggesting that these samples possess distinct volatile profiles. As shown in Figure 1 and Table S1, the response values of W1C, W3C, W6S, W5C, and W3S were relatively low for most samples, indicating that compounds associated with these sensors, such as aromatic compounds, ammonia-related compounds, hydrocarbons, short-chain alkanes, and long-chain alkanes, were present at relatively low intensities. In contrast, W5S, W1S, W1W, W2S, and W2W generated stronger responses, indicating that the compounds detected by these sensors contributed more to the differentiation of the five samples.

Among these sensors, W1S generated the highest response to HS, FG, JD, and YZ, suggesting that methyl-related volatile compounds are important contributors to the overall volatile characteristics of these samples. For PM, the W1W sensor showed the strongest response, indicating relatively high concentrations of sulfur-containing compounds, organic compounds, and terpene-related compounds. This result is consistent with the ecological function of PM as an important microbial habitat in NXB fermentation, where long-term anaerobic fermentation may promote the accumulation of diverse microbial metabolites and flavor precursors [5,10].

As shown in Figure 1B, PC1 and PC2 explained 94.1% and 3.3% of the total variance, respectively, accounting for a cumulative explained variance of 97.4%. The five samples formed distinct clusters in the PCA score plot, indicating that their overall aroma characteristics were significantly different. Notably, PM was clearly separated from the other samples, suggesting that it possessed a unique aroma profile. The relative distribution of HS and FG reflected their close contact and their similar aroma characteristics. These results provide preliminary evidence that HS may serve as a transitional matrix between the fermentation substrates and the final distilled NXB.

### 3.2. Main Physicochemical Indices of JD and YZ Baijiu Samples

The concentrations of acids, alcohols, and esters are important factors that influence the aroma quality and sensory characteristics of baijiu [25,26,27]. In this study, the total acids, esters, and major volatile compounds in the two baijiu samples, JD and YZ, were determined using conventional analysis and GC-FID (Table 3). Organic acids contribute to acidity, body, and aroma complexity and serve as precursors for ester formation during fermentation and storage [27,28]. As shown in Table 3, YZ possessed a higher total acid content of 1.20 ± 0.022 g/L than JD (0.88 ± 0.015 g/L), which may contribute to a strong acidic taste and distinct flavor balance. However, excessive acid levels may also affect the harmony of the baijiu flavor if not balanced with sufficient ester compounds.

Higher alcohols, including *n*-propanol, 2-butanol, isobutyl alcohol, *n*-butanol, and isoamyl alcohol, are common VOCs in baijiu that play important roles in aroma complexity, mouthfeel, and aftertaste [29,30]. Appropriate concentrations of higher alcohols can enhance the fullness and coordination of baijiu, whereas excessive levels may lead to pungent or fuel-like off-flavors [29]. In the present study, JD contained higher concentrations of *n-*propanol, 2-butanol, isobutyl alcohol, *n-*butanol, and isoamyl alcohol than YZ (Table 3). These results suggested that JD possesses a richer alcohol-related aroma background, which may contribute to its stronger aroma intensity and fuller flavor profile.

Esters are generally regarded as the most important aromatic compounds in baijiu, especially short- and medium-chain ethyl esters, which contribute to the fruity, sweet, and floral notes [27]. JD exhibited a higher total ester content (8.26 ± 0.032 g/L) than YZ (6.68 ± 0.024 g/L). Among the characteristic esters in baijiu, ethyl acetate, ethyl lactate, ethyl butanoate, and ethyl hexanoate are considered major contributors to the aroma quality, and their concentrations and proportions strongly affect the typical baijiu flavor style [27,31]. Ethyl caproate is one of the most important characteristic aroma compounds in NXB, and the balance between ethyl caproate and ethyl lactate is closely related to the typical aroma style and flavor coordination of the product [3]. JD contained a higher concentration of ethyl caproate than YZ, with values of 3.628 ± 0.004 and 2.312 ± 0.003 g/L, respectively. In contrast, YZ had a higher concentration of ethyl lactate. The ethyl caproate/ethyl lactate ratio in JD was also higher than that in YZ, suggesting that JD has a more prominent NXB-like ester aroma and favorable ester composition [3]. Therefore, the higher total ester content, higher ethyl caproate concentration, and more favorable ethyl caproate/ethyl lactate ratio in JD indicated its more ester-dominant and characteristic NXB aroma profile compared with YZ, which is consistent with the E-nose results.

### 3.3. VOC Detection and Differential Analysis Based on HS-SPME-GC-IMS

#### 3.3.1. Identification of Abundant VOCs by GC-IMS

HS-GC-IMS was used to further characterize the volatile fingerprints of FG, HS, PM, JD, and YZ. Owing to its high sensitivity, rapid detection, and ability to distinguish subtle differences in volatile compounds, GC-IMS has been widely applied to the analysis of baijiu and fermentation-related samples [12,23]. This technique is particularly suitable for comparing volatile fingerprints among samples within complex matrices and identifying the characteristic VOCs associated with different quality grades of baijiu [23,32]. A total of 71 VOCs, excluding the internal standard 2-methyl-3-heptanone, were detected in the five samples using HS-GC-IMS (Table S2). These VOCs were mainly esters, acids, alcohols, ketones, aldehydes, furan derivatives, and other compounds. Some compounds were detected as monomers, dimers, or trimers, as commonly observed in GC-IMS because of the different concentrations and ionization behaviors of these compounds. Among the detected VOCs, esters constitute an important class of compounds responsible for the fruity and floral aroma characteristics of NXB [3,27]. For example, ethyl lactate, ethyl hexanoate, and ethyl propanoate are generally associated with fruity, sweet, pineapple-like, and grape-like aroma notes [27,33].

In addition to common ester compounds, several VOCs with distinct aromatic properties have been identified. Limonene may contribute to the citrus- and lemon-like aroma notes, whereas 2-methoxy-3-methylpyrazine and 2,3-diethylpyrazine are associated with nutty, roasted, cocoa-like, and toasted aroma characteristics [33,34]. Although these compounds are usually present at relatively low levels, they may alter the complexity and background aromas of NXB. Therefore, the GC-IMS results indicated that the five samples contained diverse aroma-related VOCs, providing a chemical basis for further elucidation of flavor differences among HS, FG, PM, and distilled baijiu.

#### 3.3.2. Detection of Common VOCs in HS and FG

**Figure 2:**
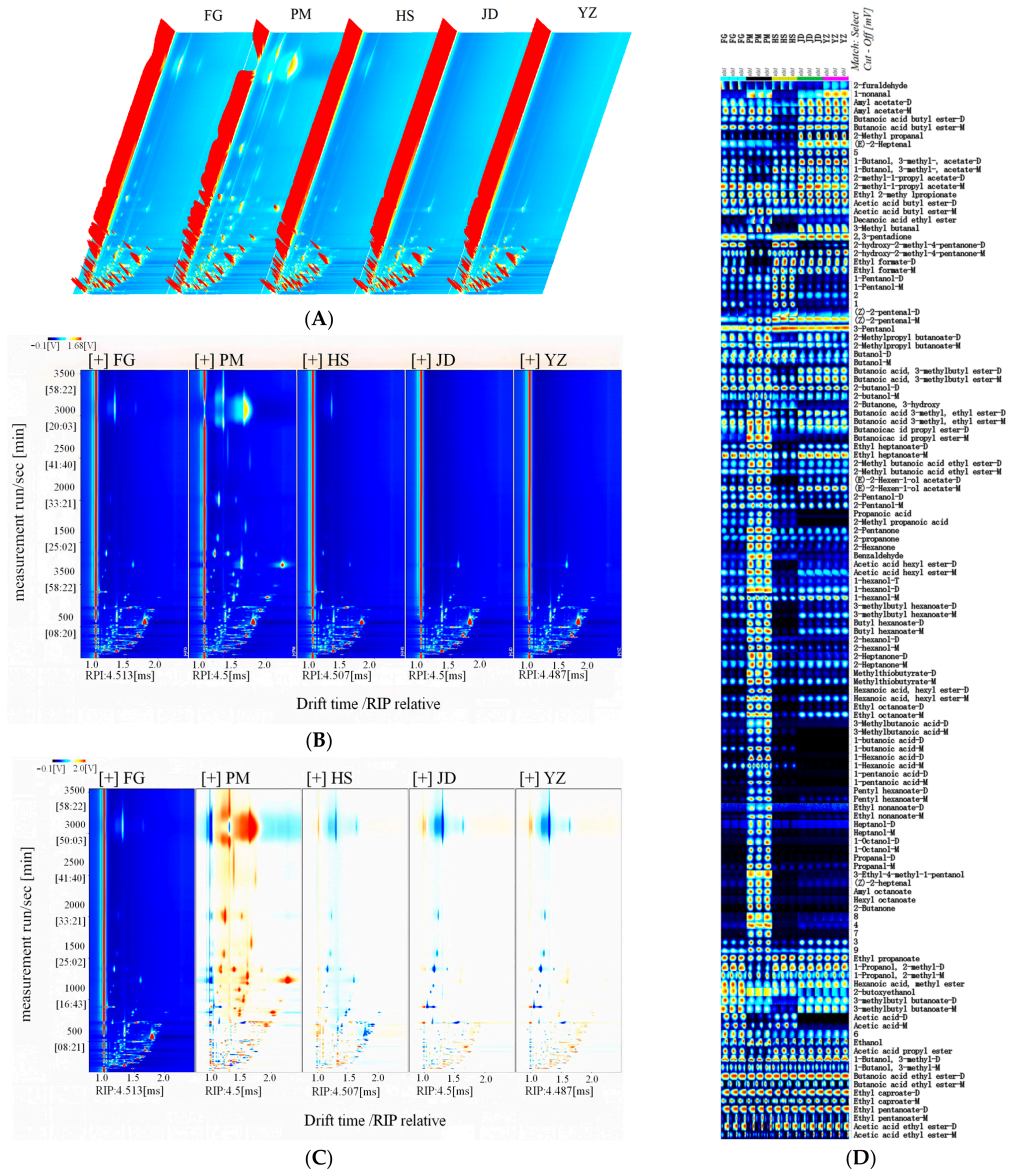
HS-GC-IMS volatile fingerprints showing the flavor relationship among FG, HS, PM, and NXB: (**A**) Three-dimensional spectrogram of VOCs. (**B**) Two-dimensional spectrogram of VOCs. (**C**) Differential spectrogram comparing VOC differences. (**D**) Fingerprint spectrogram of VOCs.

The three-dimensional GC-IMS spectra provided an intuitive overview of the VOC differences among the five samples (Figure 2A). The retention time, drift time, and signal intensity were used to characterize the position and abundance of the VOCs. The two-dimensional topographic plots further showed that most VOC signals appeared within the retention time range of 180–700 s and the drift time range of 1.0–1.7 ms (Figure 2B). These results indicated that the main volatile differences among the samples were concentrated in this region.

To more clearly visualize the differences among the samples, the FG spectrum was selected as the reference, and the spectra of HS, PM, JD, and YZ were subtracted from that of FG to generate differential plots (Figure 2C). In the differential spectra, white indicates that the target sample has a signal intensity similar to that of FG, whereas red and blue indicate higher and lower signal intensity than FG, respectively [23]. The volatile profiles of JD and YZ differed from those of FG, HS, and PM, which is mainly attributed to the enrichment of low-boiling and aroma-active compounds during distillation. By contrast, FG, HS, and PM showed similar VOC characteristics, reflecting their direct contact and metabolic interactions in the fermentation pit. Notably, HS and FG exhibited a high degree of similarity in the detected VOCs. This result is important because HS forms and accumulates at the bottom of the fermentation pit during solid-state fermentation, where it remains in direct contact with both FG and PM. In the liquid phase, HS dissolves and accumulates water-soluble organic acids, alcohols, esters, and other microbial metabolites derived from FG and PM [8,9,16]. Therefore, HS may have a stronger capacity for metabolite diffusion, migration, and exchange than solid matrices. The similarity between HS and FG suggests that HS is not only a byproduct of fermentation but also a metabolite-rich phase that reflects the flavor-related chemical state of FG.

The GC-IMS fingerprint plot further supported this interpretation (Figure 2D). In FG, compounds such as acetic acid, 3-methylbutyl butanoate, 2-butoxyethanol, methyl hexanoate, 2-methyl-1-propanol, and ethyl propanoate exhibited relatively high signal intensities. Compounds such as 1-pentanol, ethyl formate, and 2-hydroxy-2-methyl-4-pentanone were also abundant in HS. Previous studies have shown that HS is rich in organic acids and other flavor-related metabolites, and flavor compounds from FG can gradually migrate and accumulate in HS at the bottom of the fermentation pit [8,9,16]. Therefore, the presence of acids, esters, and alcohols in HS indicates that it may serve as an intermediate reservoir for flavor precursors and aroma compounds during NXB fermentation. PM contains abundant microorganisms and microbial metabolites that can provide acid- and ester-related precursors for the fermentation system. The high similarity between HS and FG volatile profiles, together with the distinct VOC characteristics of PM, suggests potential chemical relationships among these fermentation matrices. From the perspective of flavor formation, HS may represent a metabolite-rich fermentation matrix chemically associated with both PM and FG; however, whether HS directly mediates metabolite exchange within the PM–HS–FG system requires further investigation.

#### 3.3.3. Detection of Baijiu-Related VOCs in HS and FG

A GC-IMS fingerprint comparison showed that many VOCs detected in JD and YZ were also present in HS and FG, whereas some VOCs were mainly detected in PM. This distribution pattern suggests that the flavor profile of distilled NXB is closely related to the volatile and semivolatile compounds accumulated in FG, whereas the profile of HS reflects both FG-derived and PM-associated metabolic characteristics. For JD and YZ, the signal intensities of several esters, alcohols, aldehydes, and ketones were higher than those for FG, HS, and PM. This difference is reasonable because the distillation process selectively extracts and concentrates volatile compounds from the FG, resulting in higher signal intensities for the aroma-active compounds in baijiu. However, the presence of many baijiu-related VOCs in HS and FG indicates that these matrices contain important precursors or directly transferable flavor compounds before distillation.

HS is particularly important for this process because it contains both FG-derived metabolites and PM-associated compounds. Organic acids in HS can serve as substrates for ester formation, whereas alcohols may participate in esterification reactions and directly contribute to aroma formation. Esters accumulated in HS may also reflect the ongoing esterification and microbial metabolism occurring in the fermentation pit. Therefore, HS may not simply mirror the VOC profile of FG but may also participate in shaping the chemical environment that favors the formation of characteristic NXB aroma compounds. This interpretation is consistent with the role of HS as a metabolite-rich fermentation matrix chemically associated with both PM and FG. PM provides a microbial and biochemical background for acid formation and precursor generation, whereas FG serves as a direct substrate for distillation and aroma release. The proposed association of HS with PM and FG is inferred from its natural formation during fermentation and direct contact with both matrices. In this way, HS may eventually influence the aroma characteristics of distilled baijiu.

#### 3.3.4. Comparison of VOCs Between JD and YZ

**Figure 3:**
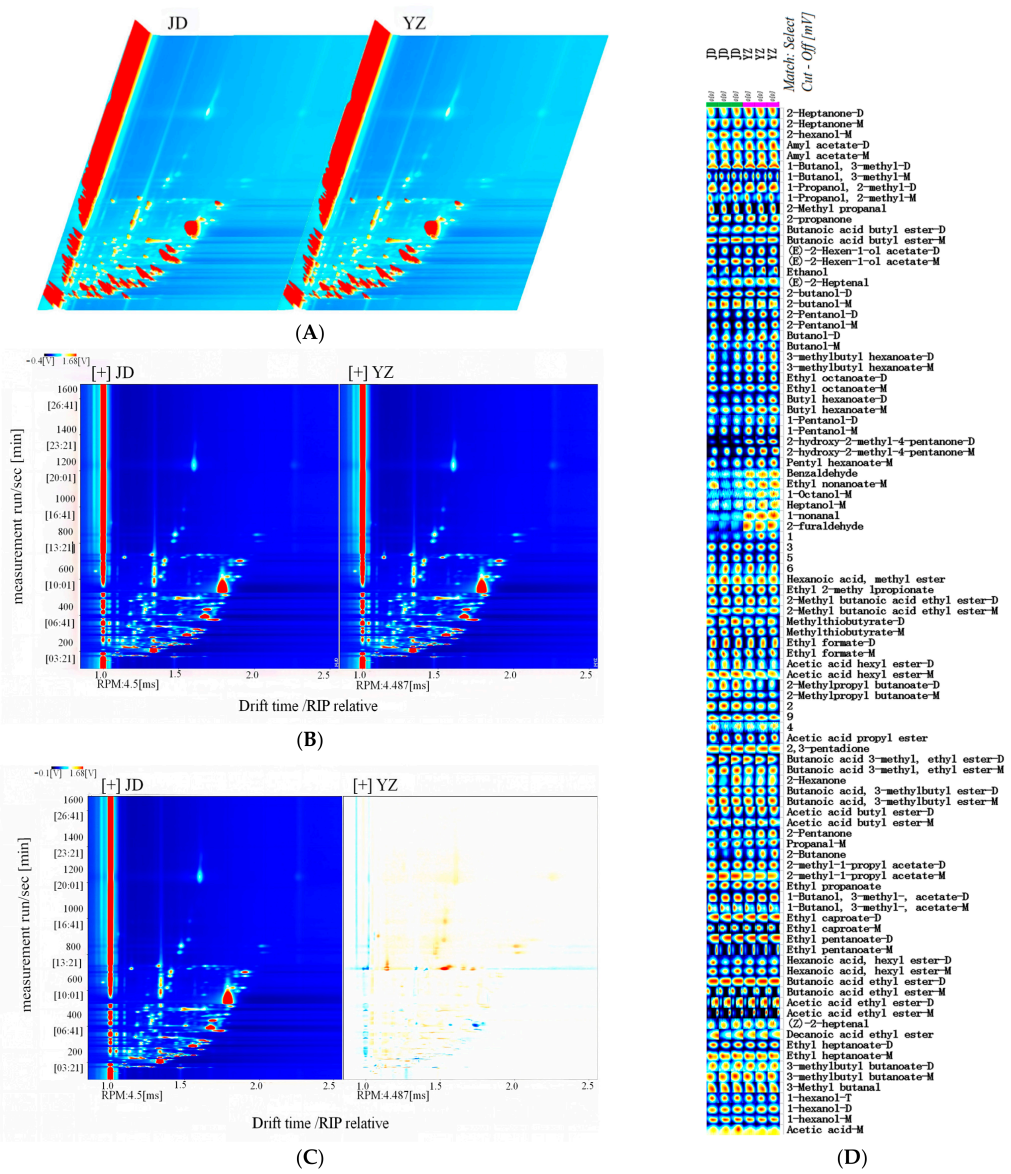
Differential GC-IMS fingerprints showing ester- and alcohol-related VOC enrichment in JD in relation to that in YZ: (**A**) Three-dimensional spectrogram of VOCs. (**B**) Two-dimensional spectrogram of VOCs. (**C**) Differential spectrogram comparing VOC differences. (**D**) Fingerprint spectrogram of VOCs.

Although ethanol concentration was standardized before volatile analysis, the complex baijiu matrix may still influence VOC release behavior. Therefore, the differences observed between JD and YZ reflect the variations in volatile profiles under standardized analytical conditions rather than the differences in VOC concentrations in the original products. As shown in Figure 3 and Table S2, several compounds, including 2-methylpropyl butanoate, hexyl acetate, ethyl formate, methyl thiobutyrate, ethyl 2-methylbutanoate, ethyl 2-methylpropionate, and methyl hexanoate, showed relatively high signal intensities in JD. In addition, other VOCs such as 2-furaldehyde, nonanal, heptanol, 1-octanol, ethyl nonanoate, benzaldehyde, pentyl hexanoate, 1-pentanol, butyl hexanoate, ethyl octanoate, 3-methylbutyl hexanoate, butanol, 2-pentanol, 2-butanol, 2-methylpropanal, 3-methyl-1-butanol, amyl acetate, 2-hexanol, and 2-heptanone were also more abundant in JD than in YZ (Table S2).

These compounds are associated with different aroma attributes, including fruity, floral, fatty, sweet, malty, and aldehyde contents. The higher relative contents of ester-related VOCs in JD were consistent with the GC-FID results, which showed that JD contained more esters and ethyl caproate than YZ. Alcohols, such as 3-methyl-1-butanol, butanol, and pentanol, may also contribute to aroma complexity and serve as important precursors for ester biosynthesis. Therefore, the higher relative contents of both esters and alcohols in JD suggest that JD has a stronger aroma formation potential and a more ester-dominant flavor profile.

Combined with the results in Section 3.1 and Section 3.2, the GC-IMS data further suggest that JD possesses a more typical NXB aroma profile than YZ. More importantly, many compounds present at higher contents in JD were also detected in HS and FG, implying that the aroma differences between JD and YZ are possibly associated with the accumulation of flavor precursors in the fermentation system. As a percolating liquid matrix, HS may exert an indirect yet vital function in flavor compound exchange between PM and FG. Overall, GC-IMS results further verify the similarities and differences in flavor profiles among HS, PM, FG, and distilled baijiu.

### 3.4. HS-SPME-GC-MS Results Supporting the Possible Role of HS as a Flavor-Related Matrix

As reported, GC-IMS and GC-MS possess distinct advantages and are suitable for different analytical needs. In practical applications, these two techniques are often used in combination, where GC-IMS enables rapid preliminary screening followed by GC-MS for accurate identification and verification [12,13,21]. Herein, HS-SPME-GC-MS and HS-GC-IMS were used as complementary profiling tools to identify the differences and similarities in VOCs among the HS, FG, PM, and NXB samples.

#### 3.4.1. Composition and Relative Abundance of Detected VOCs

**Figure 4:**
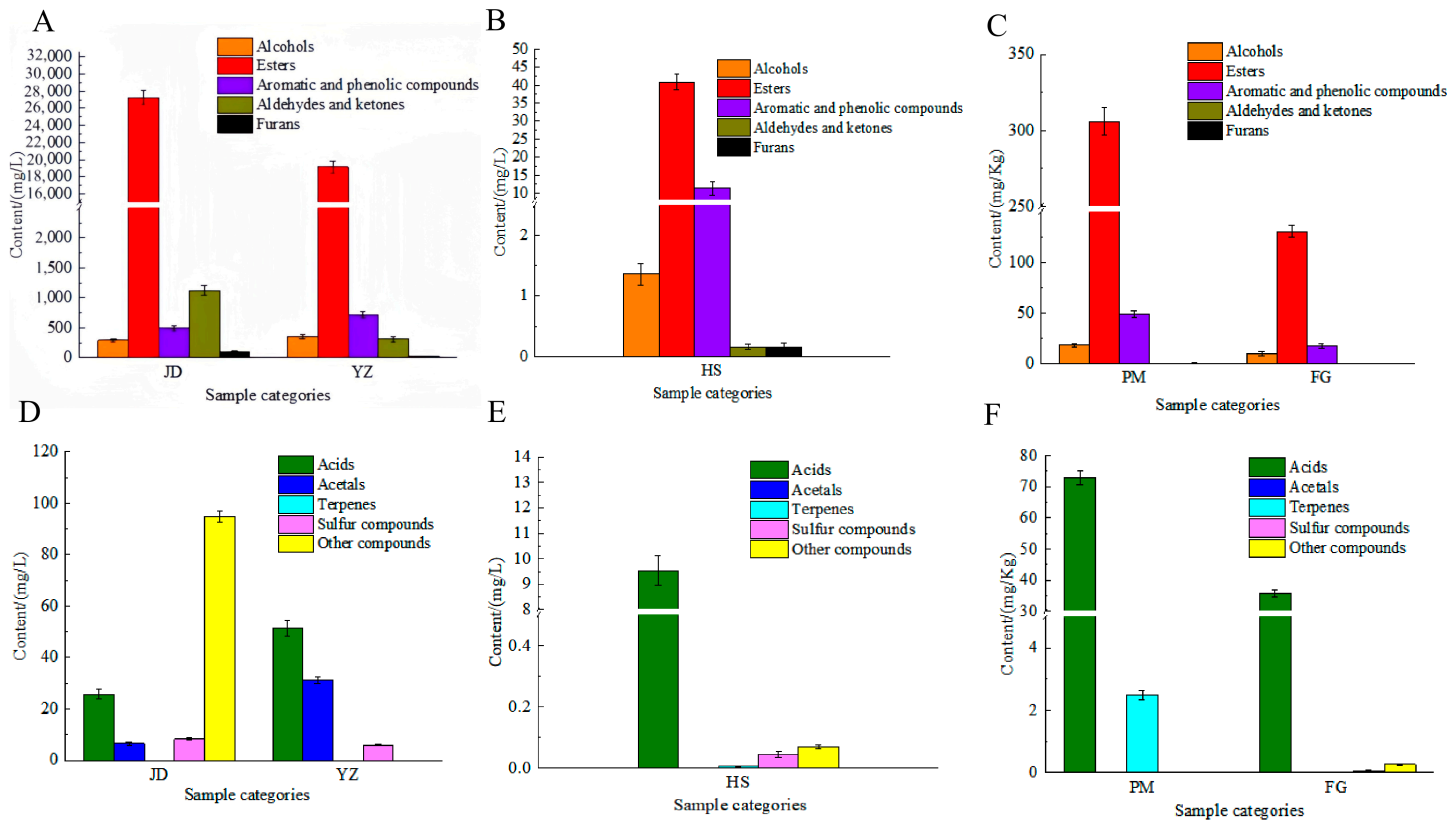
Composition and relative contents of VOC groups detected in five different samples via HS-SPME-GC-MS. Relative contents of the major VOC groups (alcohols, esters, aldehydes and ketones, aromatic and phenolic compounds, and furans) in the JD and YZ (**A**), HS (**B**), and PM and FG (**C**) samples. Relative contents of the major VOC groups (acids, acetals, terpenes, sulfur compounds, and other compounds) in the JD and YZ (**D**), HS (**E**), and PM and FG (**F**) samples.

A total of 111 VOCs were detected in YZ, including 56 esters, 5 alcohols, 7 acids, 7 aldehydes and ketones, 26 aromatics and phenols, 5 furans, 2 acetal compounds, and 3 sulfur-containing compounds (Table S3). In HS, 100 VOCs were detected, including 44 esters, 6 alcohols, 13 acids, 3 aldehydes and ketones, 23 aromatics and phenols, 1 terpene, 4 furans, 3 sulfur-containing compounds, and 3 other compounds (Table S3). Overall, esters were the most abundant volatile group in all five samples, with the highest relative ester contents observed in JD and the lowest in HS (Figure 4A,B,D,E, and Table S3). Previous studies have shown that esters usually contribute fruity, floral, sweet, and creamy aroma notes to baijiu and that their diversity and concentration are closely associated with the typical flavor characteristics of different baijiu types [3,6]. Although HS contained a lower relative ester content than distilled baijiu, it exhibited a relatively diverse volatile composition, such as esters, acids, alcohols, aromatics, and phenols. This result suggests that HS should not be regarded as a fermentation byproduct. Instead, it may serve as a metabolite-rich liquid phase that accumulates both flavor compounds and flavor precursors during fermentation. In particular, the coexistence of organic acids and alcohols in HS indicates that it may function as substrates for ester formation and participate in the establishment of an esterification-related chemical environment.

In FG and HS, esters were the predominant volatile groups, followed by acids and alcohols (Figure 4B,C,E,F), indicating that esterification and acid-related metabolism are important contributors to the aroma-active background of the fermentation system. The similarity in major VOC classes between FG and HS further supports their close metabolic relationships. Because HS forms through the gradual accumulation of liquid exudates from FG and remains in direct contact with PM at the bottom of the pit, it can dissolve, retain, and redistribute organic acids, alcohols, esters, and other microbial metabolites [8,9,16]. Therefore, HS may serve as a reservoir of flavor-associated metabolites that connects the solid-state fermentation substrate FG and the microbial habitat PM.

Compared to HS and FG, PM showed a relatively higher diversity of aromatics, phenols, and terpene-related compounds (Figure 4B,C,E,F). This result is consistent with the role of PM as a long-term microbial habitat and flavor precursor source in NXB fermentation. Previous studies have suggested that the quality and maturity of PM are closely related to the generation of key aroma precursors and flavor-active compounds in NXB [35,36]. Therefore, the VOC profile of HS, which shares characteristics with that of FG and PM, further supports its role as an ecological and metabolic transition phase in the fermentation pit [8,9,16].

#### 3.4.2. Multivariate Statistical Analysis of HS-SPME-GC-MS Results

**Figure 5:**
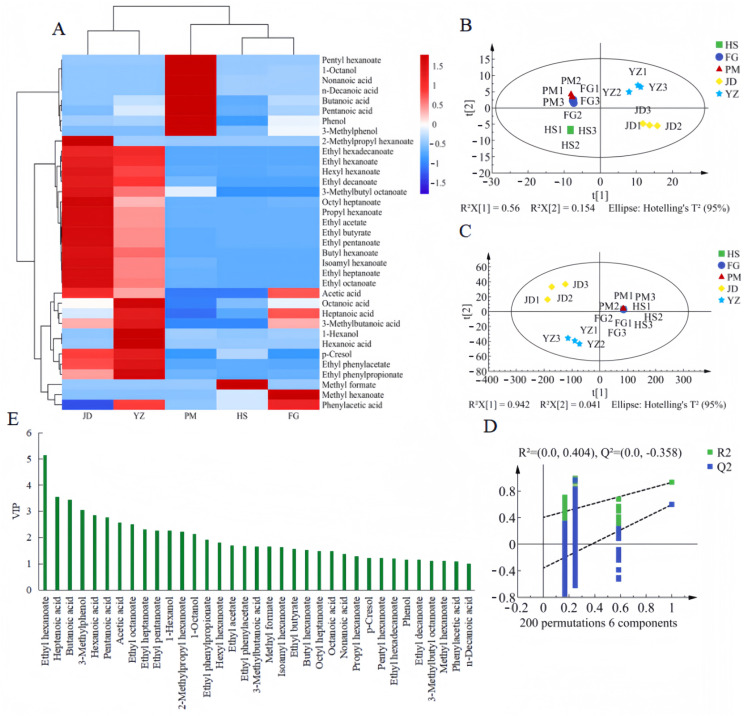
VOCs identified in five different samples via HS-SPME-GC-MS: (**A**) Content heatmap. (**B**) PCA score plot of VOCs. (**C**) Partial least squares discriminant analysis (PLS-DA) score plot of VOCs. (**D**) Confidence test results. (**E**) VIP values.

To further evaluate the differences and relationships among samples, heatmap analysis, PCA, PLS-DA, and variable importance in projection (VIP) analyses were performed based on the HS-SPME-GC-MS data (Figure 5). The statistical unit used in the analysis was the biological replicate corresponding to each independent fermentation pit, and technical analytical replicates were averaged before the construction of the multivariate data matrix. Before multivariate analysis, variables with more than 50% missing values were excluded. The remaining missing values were imputed using one-half of the minimum detected value for the corresponding variable. VOC relative content data were then log-transformed, normalized using internal-standard normalization, mean-centered, and unit-variance scaled before PCA and PLS-DA.

As shown in Figure 5A, JD and YZ generally possessed higher relative contents of many VOCs than FG, HS, and PM, mainly because of the enrichment of volatile compounds during distillation. In YZ, the relative contents of 1-hexanol, hexanoic acid, and octanoic acid were higher. In contrast, pentyl hexanoate, 1-octanol, nonanoic acid, *n-*decanoic acid, butanoic acid, pentanoic acid, phenol, and 3-methylphenol were more abundant in PM than in the other samples.

The accumulation of acids in PM and HS is important for understanding flavor interrelationships among PM, HS, FG, and distilled baijiu. Organic acids, such as butanoic acid, pentanoic acid, hexanoic acid, heptanoic acid, and octanoic acid, can directly contribute to aroma complexity and serve as precursors for ester formation [29,31]. In NXB fermentation, these acids may be produced by microbial metabolism in PM and FG and then partially accumulate in HS owing to its liquid nature. Subsequently, these acid-related precursors may be involved in ester formation in FG or contribute to the volatile profile of distilled baijiu. Thus, the distribution of acid- and ester-related compounds provides chemical evidence for the similarities and differences in VOCs among HS, FG, PM, and NXB samples.

The PCA score plot showed that the first two principal components accounted for 71.4% of the total variation (Figure 5B). FG, HS, and PM were located relatively close to each other compared with JD and YZ, indicating that their fermentation matrices shared similar volatile characteristics. This clustering trend is reasonable because FG, HS, and PM are directly connected in the fermentation pit and continuously exchange water, nutrients, microorganisms, and metabolites during fermentation. In contrast, JD and YZ were separated from the fermentation matrices, reflecting the selective concentration of aromatic compounds during distillation and the quality grade differences between the two baijiu samples.

As PLS-DA represents a supervised algorithm prone to overfitting on small datasets, we reassessed the PLS-DA model via three-fold leave-one-pit-out cross-validation. For each fold, all samples from identical biological replicate pits were withheld from model training and reserved as validation sets to eliminate information leakage among samples sourced from the same fermentation pit. The PLS-DA score plot further revealed an overlapping or close distribution among HS, FG, and PM (Figure 5C), confirming a strong metabolic association among the three fermentation-related samples. Importantly, this association does not indicate that the VOC profiles are identical. Rather, this suggests that HS may integrate chemical signals from both FG and PM. PM contributes microbial metabolites and flavor precursors; FG provides the main fermentation substrate and direct distillation matrix. Therefore, the multivariate results may also suggest that HS serves as a connecting phase within the PM–HS–FG flavor continuum.

#### 3.4.3. Identification of Key Flavor-Related Compounds via VIP Analysis

The reliability of the PLS-DA model was evaluated using 200 permutation tests (Figure 5D). In general, R^2^ and Q^2^ values closer to 1.0 indicate better model fit and predictive ability. The model exhibited an R^2^X of 0.971 and a Q^2^ of 0.809, indicating excellent explanatory and predictive performance. Moreover, the negative intercept of the Q^2^ regression line indicated that the model was reliable and showed no obvious overfitting. The Q^2^ values were calculated using a 7-fold cross-validation procedure implemented in SIMCA, which is a standard and widely accepted approach for PLS-DA model validation. The slightly higher Q^2^ values observed, compared to R^2^, can be attributed to differences in the scaling of response variance in training vs. validation folds and the relatively small dataset size. Furthermore, based on the PLS-DA model, VOCs with VIP values greater than 1 were considered important discriminatory compounds among samples [16,23].

A total of 35 VOCs with VIP > 1 were identified as the major differential metabolites contributing to sample separation. Ethyl hexanoate exhibited the highest VIP value, followed by heptenoic and butanoic acids. These compounds were closely associated with the typical aromatic characteristics of NXB [26]. Ethyl hexanoate is one of the most representative aromatic compounds in NXB and strongly contributes to its characteristic fruity and cell-like aroma. The high VIP value of ethyl hexanoate indicated that ester-related compounds were the main contributors to flavor differentiation among the samples. Notably, VIP values are interpreted only as variables contributing to group discrimination within the fitted model, not as direct evidence of biochemical importance, sensory relevance, or metabolite transfer of flavor metabolites. Further, the flavor relevance of differential metabolites should not be inferred only from relative contents or VIP values, especially for the compounds as contributors to Baijiu aroma. Therefore, future studies should combine sensory evaluation, odor activity value analysis, gas chromatography–olfactometry (GC-O) aroma recombination, or omission experiments to confirm their specific contributions to the aroma profile of NXB.

Other important VOCs include 3-methylphenol, hexanoic acid, pentanoic acid, acetic acid, ethyl octanoate, ethyl heptanoate, ethyl pentanoate, 1-hexanol, and 2-methylpropyl hexanoate (Figure 5E). These compounds can be classified into three categories. The first includes organic acids, such as acetic acid, pentanoic acid, hexanoic acid, heptenoic acid, and butanoic acid, which may contribute to sour, fatty, cheesy, or complex aroma notes and serve as important ester precursors. The second category includes esters, such as ethyl hexanoate, ethyl octanoate, ethyl heptanoate, ethyl pentanoate, and 2-methylpropyl hexanoate, which are mainly associated with fruity and floral aroma characteristics [26,27]. The third category includes alcohols and phenolic compounds, such as 1-hexanol and 3-methylphenol, which may contribute to aroma complexity and reflect microbial metabolism in the fermentation system. Notably, acids, alcohols, and esters constituted a substantial proportion of the high-VIP compounds. Organic acids accumulated in HS may provide substrates for ester biosynthesis, whereas alcohols may serve as esterification partners or aroma contributors. Esters represent the final aroma-active products that largely determine the typical flavor of NXB. Therefore, the prominent contribution of these compounds to sample discrimination suggests that HS may play an intermediate role in NXB flavor formation by connecting acid production, alcohol metabolism, and ester accumulation between PM and FG.

#### 3.4.4. Hypothesized Role of HS in NXB Fermentation System

HS-SPME-GC-MS provides strong evidence for the intermediate role of HS in the NXB fermentation system. PM containing several abundant acids, phenols, and aromatics indicates that it functions as a microbial habitat and precursor source. FG shows a volatile profile closely related to fermentation-derived esters, acids, and alcohols, which directly influence the composition of distilled baijiu. HS exhibits important chemical characteristics similar to those of both PM and FG and contains diverse esters, acids, alcohols, aromatics, and phenols.

During fermentation, PM contains microorganisms and metabolic precursors, particularly acid-related compounds. HS may accumulate soluble and semivolatile metabolites and facilitate their redistribution owing to its liquid state and direct contact with both PM and FG. FG serves as the main matrix for further transformation and distillation, promoting the accumulated flavor compounds and precursors into the distilled baijiu. The presence of shared metabolites across PM, HS, and FG suggests that HS is likely involved in aroma development throughout the fermentation matrix system. Nevertheless, we acknowledge that the above findings are based on a single production system and volatile profiling rather than direct functional or microbiological evidence. Further studies integrating metabolomics, microbial community analysis, and functional validation are required to confirm the proposed metabolite reservoir role of HS in NXB fermentation.

## 4. Conclusions

In this study, E-nose, HS-GC-IMS, and HS-SPME-GC-MS were conducted to compare the volatile profiles of HS, FG, PM, and two quality grades of NXB. The E-nose results showed that the five samples had distinct volatile fingerprints, with PM exhibiting stronger responses related to sulfur-containing, organic, and terpene-associated compounds. GC-FID showed that the JD quality grade had a higher total ester content, higher ethyl caproate concentration, and a more favorable ethyl caproate/ethyl lactate ratio than the YZ quality grade, indicating a more typical NXB aroma profile. HS-GC-IMS detected 71 VOC signals, and HS-SPME-GC-MS identified 183 volatile compounds, with esters and acids as the dominant compound classes. Multivariate analysis revealed a close relationship between HS, FG, and PM, suggesting that there exist similarities and differences in VOC fingerprints among samples. Owing to its liquid state and direct contact with FG and PM, HS may exert an indirect yet vital function in the transfer of flavor compounds between both matrices, thereby contributing to aroma formation in NXB fermentation. These findings provide useful insights into flavor regulation and quality control in NXB production; for example, HS collected after FG fermentation can be concentrated and distilled to produce a liquor with special flavor for blending with other commercial baijiu.

Several limitations of the present analysis should be acknowledged. The inferred links among the volatile profiles of HS, FG, PM, and two quality grades of NXB should be regarded as testable hypotheses that warrant further validation via time-series sampling, metabolite flux analysis, metatranscriptomics, and brewing microcosm experiments. The main conclusion of this study, i.e., HS plays a key role in aroma formation during NXB fermentation, is chemically plausible but remains inferential and requires further experimental validation. Additionally, the relatively small sample size may affect the generalizability of the results. Therefore, future studies should include larger sample sizes and broader sample sources (sampling during various periods of fermentation processes) to improve the applicability of the conclusions.

## Figures and Tables

**Table 1:** Performance description of the E-nose system.

Serial Number	Sensor Name	Performance Description
1	W1C	Sensitive to aromatic compounds (benzene derivatives)
2	W5S	Highly sensitive to nitrogen oxides
3	W3C	Sensitive to aromatic components and ammonia-containing compounds
4	W6S	Mainly selective toward hydrides
5	W5C	Sensitive to short-chain alkanes and aromatic components
6	W1S	Highly sensitive to methyl-containing compounds
7	W1W	Highly sensitive to sulfides
8	W2S	Sensitive to alcohols and aldehydes/ketones
9	W2W	Sensitive to aromatic components and organic sulfides
10	W3S	Sensitive to long-chain alkanes

**Table 2:** GC flow program.

Time	E1 (Drift Gas Flow Rate, mL/min)	E2 (Gas Flow Rate, mL/min)	R (Data Collection)
00:00,000	75.0	2.0	Recording
02:00,000	75.0	2.0	
10:00,000	75.0	10.0	
20:00,000	75.0	100.0	
30:00,000	75.0	100.0	
40:00,000	75.0	150.0	
60:00,000	75.0	150.0	Stop

**Table 3:** Results of the main physicochemical indices of JD and YZ baijiu samples (ethanol, total acids, total esters, and main VOCs detected by GC-FID).

Category	CAS	Unit	Baijiu Samples	
			JD	YZ
Ethanol	/	% ethanol by volume	77.0 ± 0.1	72.0 ± 0.1
Total acid	/	g/L	0.88 ± 0.015 ^a^	1.20 ± 0.022 ^a^
Total ester	/	g/L	8.26 ± 0.032 ^b^	6.68 ± 0.024 ^a^
n-Propyl alcohol	71-23-8	g/L	0.477 ± 0.011 ^a^	0.424 ± 0.013 ^b^
Sec-butyl alcohol	78-92-2		0.055 ± 0.002 ^a^	0.046 ± 0.001 ^b^
Isobutanol	78-83-1		0.165 ± 0.021 ^b^	0.132 ± 0.012 ^d^
n-Butanol	71-36-3		0.312 ± 0.001 ^b^	0.286 ± 0.002 ^c^
Iso-amyl alcohol	123-51-3		0.404 ± 0.002 ^c^	0.356 ± 0.002 ^c^
Ethyl acetate	141-78-6		4.485 ± 0.008 ^a^	2.683 ± 0.005 ^a^
Ethyl butyrate	105-54-4		2.263 ± 0.003 ^a^	0.165 ± 0.001 ^c^
Ethyl lactate	97-64-3		0.993 ± 0.001 ^a^	2.166 ± 0.003 ^a^
Ethyl caproate	123-66-0		3.628 ± 0.004 ^b^	2.312 ± 0.003 ^a^

Note: Means ± standard deviations (*n* = 3). Different letters in each row indicate significant differences based on Duncan’s test (*p* < 0.05).

## Data Availability

The original contributions presented in this study are included in the article/Supplementary Material. Further inquiries can be directed to the corresponding authors.

## Supplementary Materials

The following supporting information can be downloaded at https://www.mdpi.com/article/10.3390/foods15152722/s1, Figure S1: The representative chromatograms of different samples. (A) The representative chromatograms of FG; (B) The representative chromatograms of HS; (C) The representative chromatograms of PM; (D) The representative chromatograms of JD; (E) The representative chromatograms of YZ; Table S1: Responses of E-nose sensors; Table S2: The relative contents of VOCs detected in 5 different samples via HS-GC-IMS; Table S3: The relative contents of VOCs detected in 5 different samples via HS-SPME-GC-MS.

## Abbreviations

The following abbreviations are used in this manuscript:

NXB	Nongxiangxing Baijiu
FG	Fermented grains
PM	Pit mud
HS	Huangshui
VOC	volatile organic compound
GC-FID	gas chromatography–flame ionization detection
GC-MS	gas chromatography–mass spectrometry
GC×GC-TOF-MS	gas chromatography–time-of-flight mass
GC-IMS	gas chromatography–ion mobility spectrometry
E-nose	electronic nose
HS-SPME-GC-MS	headspace solid-phase microextraction–gas chromatography–mass spectrometry
ANOVA	one-way analysis of variance
SD	standard deviation
PCA	principal component analysis
PLS-DA	partial least squares-discriminant analysis
VIP	variable importance in projection
DB-FFAP	DB-free fatty acid phase
Dt	drift time
DVB/CAR/PDMS	divinylbenzene/carboxen/polydimethylsiloxane
HS-SPME	headspace solid-phase microextraction
ITEX	in-tube extraction
JD	special-grade Nongxiangxing Baijiu
RIP	reaction ion peak
Rt	retention time
YZ	superior-grade Nongxiangxing Baijiu
RI	retention index
