# Supplementary material for: Comparative Volatile Profiling Reveals the Chemical Association of Huangshui with Fermented Grains, Pit Mud, and Nongxiangxing Baijiu
Source: Foods. 2026 Aug 2;15(15):2722. doi: 10.3390/foods15152722 (PMC13463836; doi:10.3390/foods15152722)
Supplement: Supplementary file 1 [file foods-15-02722-s001.zip › Figure-S1.pdf]

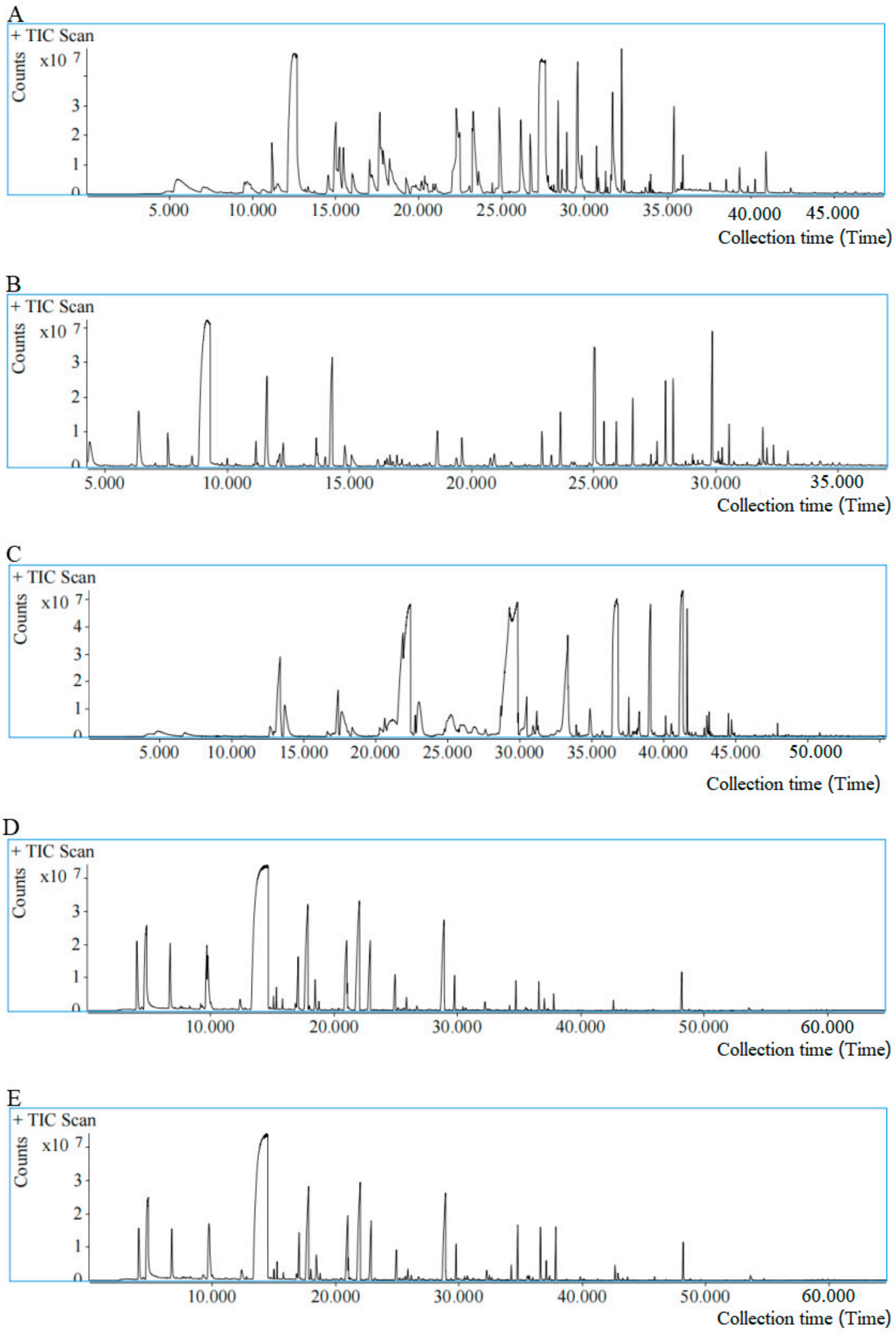

Figure S1. The representative chromatograms of different samples. (A) The representative chromatograms of FG; (B) The representative chromatograms of HS;

(C) The representative chromatograms of PM; (D) The representative chromatograms of JD; (E) The representative chromatograms of YZ.
