# Supplementary material for: Comparative Volatile Profiling Reveals the Chemical Association of Huangshui with Fermented Grains, Pit Mud, and Nongxiangxing Baijiu
Source: Foods. 2026 Aug 2;15(15):2722. doi: 10.3390/foods15152722 (PMC13463836; doi:10.3390/foods15152722)
Supplement: Supplementary file 1 [file foods-15-02722-s001.zip › Table-S1.pdf]

Table S1. Responses of E-nose sensors.

Number	Sensor Name	Performance Description	Response value of the electronic nose sensor				
			HS	PM	FG	JD	YZ
1	W1C	Sensitive to benzene derivatives and aromatic components	0.1931±0.0132	0.4820±0.0192	0.2647±0.0346	0.1251±0.0123	0.1394±0.0101
2	W5S	High sensitivity, highly sensitive to nitrogen oxides	108.7660±6.0921	31.8244±4.7485	76.6980±6.7440	176.6907±0.5954	134.571±5.3986
3	W3C	Sensitive to ammonia and aromatic components	0.4182±0.0138	0.6356±0.0148	0.4765±0.0283	0.3594±0.0123	0.3736±0.0093
4	W6S	Mainly selective for hydrides	4.7900±1.5547	1.7229±0.0747	3.3815±0.5986	4.7810±0.1426	4.7668±0.0710
5	W5C	Sensitive to short-chain alkanes and aromatic components	0.4315±0.0115	0.6268±0.0157	0.4804±0.0231	0.3719±0.0138	0.3852±0.0090
6	W1S	Sensitive to methane and methyl compounds	133.9419±10.7100	34.6687±2.3028	101.5464±20.2463	177.0697±4.0667	170.9175±9.9530
7	W1W	Sensitive to sulfur compounds, organic compounds, and terpenoids	123.2459±7.5503	75.4812±3.1345	98.1285±5.2628	140.6969±4.3029	127.6228±1.2372
8	W2S	Sensitive to alcohols and aldehydes/ketones	56.9037±16.7732	13.0845±0.7569	34.6124±6.0073	62.6225±3.2820	59.4336±2.1356
9	W2W	Sensitive to compounds with aromatic ring structures, sulfur-containing organic compounds	45.2236±5.2023	24.5497±0.9017	34.2231±1.6659	50.9955±0.6831	48.1034±0.8674
10	W3S	Sensitive to long-chain alkanes	1.7541±0.0779	1.3278±0.0143	1.6089±0.1081	2.3560±0.1461	2.2425±0.0854

Annotation: E-nose, electronic nose; JD, special grades of NXB, 77.0 ± 0.1% ethanol by volume; YZ, superior grades of NXB, 72.0± 0.1% ethanol by volume; NXB, Nongxiangxing Baijiu; HS, Huangshui; FG, Fermented grains; PM, Pit mud.
