# Supplementary material for: Comparative Volatile Profiling Reveals the Chemical Association of Huangshui with Fermented Grains, Pit Mud, and Nongxiangxing Baijiu
Source: Foods. 2026 Aug 2;15(15):2722. doi: 10.3390/foods15152722 (PMC13463836; doi:10.3390/foods15152722)
Supplement: Supplementary file 1 [file foods-15-02722-s001.zip › Table-S2.pdf]

Table S2. The relative contents of VOCs detected in 5 different samples via HS-GC-IMS.

Compound	CAS	Formula	MW	RI	Rt [sec]	Dt [a.u.]	FG / ($mg \cdot Kg^{-1}$)	PM / ($mg \cdot Kg^{-1}$)	HS / ($mg \cdot L^{-1}$)	JD / ($mg \cdot L^{-1}$)	YZ / ($mg \cdot L^{-1}$)	Comment
**Alcohols**												
1-Butanol, 3-methyl-M	123-51-3	C5H12O	88.1	1212.6	492.524	1.23785	0.4921±0.0110	0.5009±0.0607	0.2786±0.0070	2.0032±0.0309	1.7539±0.0229	whiskey, banana, fruity
1-Butanol, 3-methyl-D	123-51-3	C5H12O	88.1	1213.9	494.499	1.49379	1.6047±0.0480	1.1919±0.1401	0.9176±0.0176	7.1864±0.1410	7.4789±0.1199	whiskey, banana, fruity
3-Ethyl-4-methyl-1-pentanol	U144071	C8H18O	130.2	1524.7	1007.258	1.43173	n.d.	0.0772±0.0161	n.d.	n.d.	n.d.	null
1-Propanol, 2-methyl-M	78-83-1	C4H10O	74.1	1095.6	336.177	1.17468	0.2066±0.0026	0.1664±0.0218	0.1108±0.0047	0.8621±0.0457	0.7697±0.0483	fresh, alcoholic, leather
1-Propanol, 2-methyl-D	78-83-1	C4H10O	74.1	1096.9	337.679	1.36865	0.6126±0.0168	0.2484±0.0301	0.3252±0.0074	2.6199±0.1068	2.7604±0.0195	fresh, alcoholic, leather
Ethanol	64-17-5	C2H6O	46.1	943.1	218.63	1.14483	1.3506±0.0064	1.5594±0.1949	0.6210±0.0137	5.3455±0.1758	5.7685±0.0375	aromaticity
2-butanol-D	78-92-2	C4H10O	74.1	1028.7	276.246	1.31755	0.8500±0.0382	0.9477±0.1157	0.4815±0.0043	2.9775±0.0331	3.2635±0.0082	fruity
2-butanol-M	78-92-2	C4H10O	74.1	1029.3	276.731	1.14911	0.1419±0.0025	0.1650±0.0238	0.0650±0.0013	0.6460±0.0336	0.6484±0.0051	fruity
2-Pentanol-M	6032-29-7	C5H12O	88.1	1122.7	368.278	1.21472	0.2308±0.0032	0.2566±0.0297	0.1177±0.0027	0.7445±0.0049	0.7678±0.0232	Fusel Oil, Green
2-Pentanol-D	6032-29-7	C5H12O	88.1	1124.8	370.918	1.43417	0.2277±0.0017	0.4295±0.0553	0.0939±0.0005	0.4694±0.0189	0.5446±0.0358	Fusel Oil, Green
Butanol-D	71-36-3	C4H10O	74.1	1150.7	404.693	1.37699	0.3621±0.0149	0.4103±0.0407	0.2242±0.0057	1.0690±0.0143	1.2480±0.0312	wine
Butanol-M	71-36-3	C4H10O	74.1	1150.9	404.954	1.17649	0.2653±0.0065	0.2841±0.0312	0.1463±0.0069	1.0067±0.0135	1.0435±0.0150	wine
2-hexanol-M	626-93-7	C6H14O	102.2	1232.2	522.397	1.27808	0.1483±0.0053	0.3020±0.0378	0.0695±0.0020	0.4279±0.0090	0.4420±0.0073	fruity, fat, cauliflower
1-Pentanol-M	71-41-0	C5H12O	88.1	1265.5	577.117	1.25016	0.1225±0.0039	0.1479±0.0243	0.1198±0.0033	0.2920±0.0063	0.3455±0.0041	balsamic
1-hexanol-D	111-27-3	C6H14O	102.2	1371.9	736.949	1.6402	0.1999±0.0067	0.3175±0.0569	0.0813±0.0024	0.9032±0.0461	0.9635±0.0129	fresh, fruity, wine, sweet, green
1-hexanol-T	111-27-3	C6H14O	102.2	1371.6	736.459	1.98576	0.0499±0.0031	0.1590±0.0221	0.0243±0.0004	0.1286±0.0035	0.1408±0.0107	fresh, fruity, wine, sweet, green
1-hexanol-M	111-27-3	C6H14O	102.2	1371.9	736.949	1.32562	0.4440±0.0191	0.5505±0.0701	0.2005±0.0045	2.8642±0.1112	2.7206±0.0394	fresh, fruity, wine, sweet, green
Heptanol-M	53535-33-4	C7H16O	116.2	1469.5	899.752	1.39925	0.03220±0.0015	0.3040±0.0509	0.0125±0.0010	0.1225±0.0111	0.1451±0.0062	herb
1-Octanol-M	111-87-5	C8H18O	130.2	1578.1	1123.57	1.47041	0.0549±0.0061	0.9152±0.1634	n.d.	n.d.	0.3069±0.0087	citrus, sweet, herbs, waxy, rose, mushroom
1-Octanol-D	111-87-5	C8H18O	130.2	1574.8	1115.997	1.88376	n.d.	0.1220±0.0323	n.d.	n.d.	n.d.	citrus, sweet, herbs, waxy, rose, mushroom
Heptanol-D	53535-33-4	C7H16O	116.2	1469.7	900.049	1.75938	n.d.	0.0705±0.0157	n.d.	n.d.	n.d.	herb
1-Pentanol-D	71-41-0	C5H12O	88.1	1264.3	574.97	1.50955	0.0359±0.0014	0.0607±0.0095	0.0439±0.0003	0.0796±0.0087	0.0976±0.0013	balsamic
2-hexanol-D	626-93-7	C6H14O	102.2	1231.2	520.778	1.56807	0.0562±0.0043	0.2186±0.0276	n.d.	n.d.	n.d.	fruity, fat, cauliflower
3-Pentanol	584-02-1	C5H12O	88.1	1113.4	356.905	1.20186	n.d.	0.0310±0.0051	n.d.	n.d.	n.d.	null
2-butoxyethanol	111-76-2	C6H14O2	118.2	1437.8	843.214	1.20192	0.0172±0.0010	n.d.	0.0095±0.0009	n.d.	n.d.	pungent, ether
**Acids**												
Acetic acid-D	64-19-7	C2H4O2	60.1	1478.2	916.006	1.15607	0.6377±0.0525	0.1342±0.0630	0.2694±0.0207	n.d.	n.d.	spicy

Acetic acid-M	64-19-7	C2H4O2	60.1	1478.6	916.703	1.05211	1.7796±0.0793	0.9771±0.2481	0.9221±0.0394	1.7942±0.0778	1.7478±0.0149	spicy
1-Hexanoic acid-D	142-62-1	C6H12O2	116.2	2051	2956.639	1.65994	2.8509±0.2611	30.9231±4.6102	n.d.	n.d.	n.d.	sour, fatty, cheese, pungent, Daqu liquor
1-Hexanoic acid-M	142-62-1	C6H12O2	116.2	2043.9	2914.141	1.30222	7.1951±0.5156	11.1351±1.3254	1.9078±0.1492	n.d.	n.d.	sour, fatty, cheese, pungent, Daqu liquor
1-pentanoic acid-D	109-52-4	C5H10O2	102.1	1861	2004.67	1.51447	n.d.	0.7375±0.2176	n.d.	n.d.	n.d.	fatty, decay, slightly sour, slightly sweet
1-pentanoic acid-M	109-52-4	C5H10O2	102.1	1859.4	1998.136	1.23609	0.7422±0.0747	4.3112±0.7497	0.2008±0.0105	n.d.	n.d.	fatty, decay, slightly sour, slightly sweet
3-Methylbutanoic acid-M	503-74-2	C5H10O2	102.1	1735.7	1551.319	1.21642	0.4703±0.0574	1.5661±0.3460	0.1126±0.0054	n.d.	n.d.	sour, foot sweat, cheese
3-Methylbutanoic acid-D	503-74-2	C5H10O2	102.1	1734.8	1548.258	1.48116	n.d.	0.1529±0.0528	n.d.	n.d.	n.d.	sour, foot sweat, cheese
1-butanoic acid-M	107-92-6	C4H8O2	88.1	1674.4	1368.229	1.16556	1.5287±0.1273	3.2343±0.6437	0.3519±0.0218	n.d.	n.d.	strong acetic acid, cheese, butter, fruity
1-butanoic acid-D	107-92-6	C4H8O2	88.1	1672.6	1363.331	1.38074	0.2063±0.0264	1.5989±0.6580	0.0351±0.0026	n.d.	n.d.	strong acetic acid, cheese, butter, fruity
2-Methyl propanoic acid	79-31-2	C4H8O2	88.1	1596.7	1167.326	1.15974	0.1803±0.0142	0.2894±0.0615	0.0492±0.0035	n.d.	n.d.	yogurt, rancid cream
Propanoic acid	79-09-4	C3H6O2	74.1	1563.4	1090.332	1.11369	0.2144±0.0246	0.3619±0.0841	0.0578±0.0054	n.d.	n.d.	yogurt, vinegar
**Esters**												
Butanoic acid ethyl ester-D	105-54-4	C6H12O2	116.2	1046.5	290.735	1.56263	2.4328±0.0734	2.1426±0.2739	1.3926±0.0142	10.9456±0.1676	10.5909±0.0958	pineapple, fruity, ester, whiskey
Acetic acid propyl ester	109-60-4	C5H10O2	102.1	990.5	247.547	1.47988	0.3773±0.0238	0.1800±0.0279	0.2230±0.0046	1.7069±0.0322	1.7187±0.0217	fruity, pear
Acetic acid ethyl ester-D	141-78-6	C4H8O2	88.1	912.9	202.642	1.33682	3.8313±0.2437	2.0003±0.2636	2.5544±0.0665	19.5794±0.4953	18.7730±0.1288	fresh, fruity, sweet, grassy
Acetic acid ethyl ester-M	141-78-6	C4H8O2	88.1	912.1	202.303	1.09529	0.3173±0.0043	0.2085±0.0228	0.1513±0.0060	1.0153±0.0200	0.9458±0.0037	fresh, fruity, sweet, grassy
2-Methyl butanoic acid ethyl ester-M	7452-79-1	C7H14O2	130.2	1063.8	305.633	1.24684	0.0984±0.0010	0.1390±0.0185	0.0361±0.0014	0.3914±0.0068	0.3302±0.0051	apple
Butanoic acid 3-methyl, ethyl ester-M	108-64-5	C7H14O2	130.2	1080.9	320.964	1.26115	0.1392±0.0034	0.1792±0.0231	0.0580±0.0044	0.5894±0.0174	0.5538±0.0170	apple, banana, sour and sweet
Butanoic acid 3-methyl, ethyl ester-D	108-64-5	C7H14O2	130.2	1080.9	320.964	1.65105	0.3405±0.0220	0.5928±0.1108	0.1083±0.0049	2.1070±0.1931	2.0977±0.0493	apple, banana, sour and sweet
2-Methyl butanoic acid ethyl ester-D	7452-79-1	C7H14O2	130.2	1063.5	305.338	1.64628	0.1555±0.0066	0.2835±0.0421	0.0454±0.0032	0.6684±0.0259	0.6462±0.0173	apple
Butanoic acid ethyl ester-M	105-54-4	C6H12O2	116.2	1043.6	288.375	1.20424	0.2150±0.0019	0.2184±0.0277	0.1181±0.0035	0.9704±0.0275	0.9157±0.0088	pineapple, fruity, ester, whiskey
Hexanoic acid, methyl ester	106-70-7	C7H14O2	130.2	1193	464.485	1.28447	0.1256±0.0034	0.0748±0.0093	0.0528±0.0013	0.5028±0.0156	0.4590±0.0041	pineapple, apricot, fruity
Acetic acid butyl ester-D	123-86-4	C6H12O2	116.2	1087.5	327.176	1.62389	0.5459±0.0285	0.3957±0.0555	0.2707±0.0160	2.7225±0.0864	2.8251±0.0916	fruity

Acetic acid butyl ester-M	123-86-4	C6H12O2	116.2	1087.7	327.342	1.23662	0.1532±0.0019	0.1604±0.0176	0.0836±0.0025	0.6150±0.0087	0.5458±0.0117	fruity
Butanoic acid butyl ester-D	109-21-7	C8H16O2	144.2	1226.1	512.808	1.80545	0.4670±0.0204	0.5244±0.0692	0.0825±0.0031	2.7438±0.0520	2.9974±0.0268	fruity, banana, pineapple, green cherry , tropical fruit mature and juicy fruity aroma
Butanoic acid butyl ester-M	109-21-7	C8H16O2	144.2	1225	511.21	1.33807	0.2866±0.0082	0.2569±0.0319	0.0938±0.0005	1.4448±0.0728	1.3583±0.0113	fruity, banana, pineapple, green cherry , tropical fruit mature and juicy fruity aroma
Acetic acid hexyl ester-D	142-92-7	C8H16O2	144.2	1301.6	638.199	1.89771	0.0167±0.0018	0.0515±0.0077	n.d.	0.0749±0.0054	0.0623±0.0068	fruity, green, apple, banana, sweet
Acetic acid hexyl ester-M	142-92-7	C8H16O2	144.2	1300.9	637.26	1.40612	0.0879±0.0047	0.1499±0.0204	0.0126±0.0007	0.3831±0.0104	0.3427±0.0165	fruity, green, apple, banana, sweet
Butanoic acid, 3-methylbutyl ester-D	109-19-3	C9H18O2	158.2	1287.9	617.075	1.89963	0.1684±0.0067	0.2809±0.0330	0.0365±0.0002	1.1705±0.0372	1.1902±0.0299	bananas, blue cheese
Butanoic acid, 3-methylbutyl ester-M	109-19-3	C9H18O2	158.2	1287.1	615.666	1.39076	0.2463±0.0072	0.2894±0.0369	0.1052±0.0031	1.2933±0.0196	1.2427±0.0064	bananas, blue cheese
Hexanoic acid, hexyl ester-D	6378-65-0	C12H24O2	200.3	1625.4	1237.923	2.27448	0.2730±0.0204	5.6777±0.8274	n.d.	1.7148±0.2484	1.7349±0.0328	herb, vegetable and fruity
Hexanoic acid, hexyl ester-M	6378-65-0	C12H24O2	200.3	1625.1	1237.018	1.60279	0.9354±0.0823	2.4984±0.3044	0.2399±0.0087	5.9102±0.6717	5.8461±0.1529	herb, vegetable and fruity
Decanoic acid ethyl ester	110-38-3	C12H24O2	200.3	1665	1342.169	1.61365	n.d.	n.d.	0.0285±0.0021	0.6093±0.0811	0.5864±0.0494	fruity, wine, pear, brandy
Butanoicac id propyl ester-D	105-66-8	C7H14O2	130.2	1128.6	375.581	1.68554	n.d.	0.1716±0.0275	n.d.	n.d.	n.d.	pineapple, almond, sweet, banana, fruity
Butanoicac id propyl ester-M	105-66-8	C7H14O2	130.2	1130	377.403	1.27248	0.0179±0.0003	0.0520±0.0067	0.0078±0.0004	n.d.	n.d.	pineapple, almond, sweet, banana, fruity
1-Butanol, 3-methyl-, acetate-D	123-92-2	C7H14O2	130.2	1131.4	379.226	1.74901	0.6780±0.0243	0.2751±0.0535	0.3494±0.0077	5.5849±0.2192	5.4352±0.1360	sweet, banana, fruity
Ethyl 2-methy lpropionate	97-62-1	C6H12O2	116.2	981.4	241.538	1.56534	0.3225±0.0197	0.4022±0.0668	0.1791±0.0019	1.9217±0.0401	1.8337±0.0228	sweet, fruity, alcoholic, rummy
1-Butanol, 3-methyl-, acetate-M	123-92-2	C7H14O2	130.2	1134.1	382.616	1.30536	0.2031±0.0061	0.0985±0.0102	0.1164±0.0023	0.9605±0.0072	0.8936±0.0176	sweet, banana, fruity
2-methyl-1-propyl acetate-D	110-19-0	C6H12O2	116.2	1023.2	271.985	1.61112	0.1952±0.0127	0.0582±0.0094	0.1087±0.0012	1.5158±0.0215	1.5231±0.0201	fruity, raw pear and raspberrie
2-methyl-1-propyl acetate-M	110-19-0	C6H12O2	116.2	1021.7	270.8	1.22995	0.0695±0.0023	0.0618±0.0071	0.0262±0.0004	0.3409±0.0120	0.2934±0.0017	fruity, raw pear and raspberrie
2-Methylpropyl butanoate-D	539-90-2	C8H16O2	144.2	1163.4	422.274	1.80041	0.0387±0.002	0.0393±0.0158	0.0084±0.0007	0.1701±0.0170	0.1250±0.0222	apple,pineapple
2-Methylpropyl butanoate-M	539-90-2	C8H16O2	144.2	1163	421.652	1.33479	0.1252±0.0055	0.1259±0.0368	0.0623±0.0016	0.5213±0.0254	0.4337±0.0452	apple,pineapple
Methylthiobutyrate-D	2432-51-1	C5H10OS	118.2	1206.5	483.585	1.51398	0.0296±0.0020	0.2477±0.0589	0.0113±0.0004	0.1967±0.0165	0.1816±0.0090	Cabbage, Cheese, Garlic, Sulfur
Methylthiobutyrate-M	2432-51-1	C5H10OS	118.2	1205.8	482.671	1.18457	0.1614±0.0063	0.3479±0.0471	0.0789±0.0017	0.7885±0.0048	0.7230±0.0202	Cabbage, Cheese, Garlic, Sulfur
3-methylbutyl butanoate-M	106-27-4	C9H18O2	158.2	1277.6	598.297	1.4042	0.0651±0.0041	0.0446±0.0057	0.0097±0.0003	0.2477±0.0052	0.2536±0.0137	bananas, pears
3-methylbutyl butanoate-D	106-27-4	C9H18O2	158.2	1277.3	597.828	1.92651	0.0310±0.0015	0.0176±0.0021	n.d.	0.1235±0.0151	0.1157±0.0017	bananas, pears
3-methylbutyl hexanoate-M	2198-61-0	C11H22O2	186.3	1461.3	884.889	1.52952	0.2100±0.0225	0.3123±0.0459	0.0164±0.0003	0.9394±0.1427	1.0731±0.0028	apple, pineapple, fruity
3-methylbutyl hexanoate-D	2198-61-0	C11H22O2	186.3	1461.2	884.552	2.13873	0.0260±0.0056	0.0986±0.0228	n.d.	0.1185±0.0295	0.1433±0.0065	apple, pineapple, fruity

Ethyl caproate-D	123-66-0	C8H16O2	144.2	1243.2	539.764	1.7979	9.9289±0.2982	9.8760±1.2252	4.6549±0.0511	48.3254±0.4954	47.5919±0.2131	pineapple, fruity, wine
Ethyl caproate-M	123-66-0	C8H16O2	144.2	1241.2	536.637	1.33646	1.7572±0.0427	1.5875±0.2182	1.0020±0.0313	7.9043±0.0646	7.5409±0.0164	pineapple, fruity, wine
Ethyl propanoate	105-37-3	C5H10O2	102.1	973.5	236.656	1.45275	0.6903±0.0362	0.4891±0.0632	0.3786±0.0021	2.7666±0.0130	2.7210±0.0117	grape, pineapple, fruity, rum
Ethyl formate-D	109-94-4	C3H6O2	74.1	850.4	179.303	1.22273	0.1361±0.0232	0.0164±0.0034	0.1594±0.0034	1.0737±0.0212	0.9657±0.0097	spicy and stimulating taste, pineapple, rum
Ethyl pentanoate-D	539-82-2	C7H14O2	130.2	1141.3	392.066	1.67192	2.7524±0.0833	2.3554±0.2561	1.4992±0.0212	13.0686±0.1271	12.8899±0.1785	apple, pineapple, green
Ethyl pentanoate-M	539-82-2	C7H14O2	130.2	1142.6	393.769	1.27042	0.1084±0.0013	0.1130±0.0122	0.0649±0.0006	0.4033±0.0117	0.3730±0.0066	apple, pineapple, green
Ethyl formate-M	109-94-4	C3H6O2	74.1	849.8	179.101	1.06904	0.1434±0.0089	0.0595±0.0091	0.0881±0.0012	0.6186±0.0064	0.6368±0.0085	spicy and stimulating taste, pineapple, rum
Ethyl heptanoate-D	106-30-9	C9H18O2	158.2	1348.9	703.03	1.91179	1.0890±0.0671	1.9293±0.2708	0.1519±0.0073	5.2621±0.1782	5.0984±0.0560	pineapple, fruity
Ethyl heptanoate-M	106-30-9	C9H18O2	158.2	1351.4	706.597	1.40592	0.4983±0.0104	0.4793±0.0583	0.1768±0.0035	2.5837±0.0348	2.4059±0.0263	pineapple, fruity
Ethyl octanoate-D	106-32-1	C10H20O2	172.3	1440.2	847.395	2.01421	0.2317±0.0367	2.2846±0.3642	0.0231±0.0013	1.1397±0.1923	1.3800±0.0478	fruity, pineapple, apple, brandy
Ethyl octanoate-M	106-32-1	C10H20O2	172.3	1441.6	849.76	1.4778	0.5869±0.0535	1.0498±0.1383	0.1236±0.0016	2.8274±0.2035	3.0386±0.0426	fruity, pineapple, apple, brandy
Ethyl nonanoate-M	123-29-5	C11H22O2	186.3	1544	1047.866	1.54881	0.0758±0.0015	0.4485±0.0852	n.d.	0.3358±0.0213	0.4004±0.0111	banana, grape, fruity, wine, rose, fatty, waxy
Amyl acetate-D	628-63-7	C7H14O2	130.2	1179	444.985	1.75395	0.0747±0.0027	0.0523±0.0111	0.0288±0.0013	0.4627±0.0152	0.4709±0.0140	bananas, apples, pears
Amyl acetate-M	628-63-7	C7H14O2	130.2	1178.3	444.052	1.30908	0.1021±0.0038	0.1081±0.0122	0.0324±0.0004	0.4862±0.0036	0.5034±0.0170	bananas, apples, pears
(E)-2-Hexen-1-ol acetate-D	2497-18-9	C8H14O2	142.2	1333.8	681.628	1.90294	0.2299±0.0170	0.6971±0.1167	0.0175±0.0006	1.5426±0.0846	1.6306±0.0342	sweet,apple peel, banana peel
(E)-2-Hexen-1-ol acetate-M	2497-18-9	C8H14O2	142.2	1333.8	681.628	1.40238	0.3572±0.0150	0.4594±0.0639	0.0764±0.0019	1.9109±0.0473	1.9060±0.0268	sweet,apple peel, banana peel
Pentyl hexanoate-D	540-07-8	C11H22O2	186.3	1516.9	991.402	2.14968	n.d.	0.3529±0.1144	n.d.	n.d.	n.d.	Fruity, Floral, Fresh
Pentyl hexanoate-M	540-07-8	C11H22O2	186.3	1517.2	992.022	1.53541	0.1697±0.0207	1.0979±0.2071	0.0366±0.0015	0.9774±0.1559	1.1345±0.0122	Fruity, Floral, Fresh
Butyl hexanoate-D	626-82-4	C10H20O2	172.3	1419.2	811.72	2.0189	0.0626±0.0108	0.4927±0.1020	n.d.	0.4913±0.0603	0.6198±0.0075	fruity
Butyl hexanoate-M	626-82-4	C10H20O2	172.3	1419.9	812.907	1.46321	0.2130±0.02400	0.5251±0.0731	0.0276±0.0005	1.2672±0.0764	1.4910±0.0204	fruity
Amyl octanoate	638-25-5	C13H26O2	214.3	1772.1	1671.025	1.66506	n.d.	1.1936±0.1305	n.d.	n.d.	n.d.	floral
Hexyl octanoate	1117-55-1	C14H28O2	228.4	1957.6	2442.406	1.73864	n.d.	1.1210±0.1793	n.d.	n.d.	n.d.	null
Ethyl nonanoate-D	123-29-5	C11H22O2	186.3	1543.1	1045.973	2.12868	n.d.	0.0632±0.0189	n.d.	n.d.	n.d.	banana, grape, fruity, wine, rose, fatty, waxy
**Aldehydes**												
Benzaldehyde	100-52-7	C7H6O	106.1	1513.9	985.408	1.15548	0.0169±0.0008	0.0424±0.0040	0.0113±0.0006	0.0491±0.0044	0.0639±0.0062	bitter almond, cherry, nutty
2-Methyl propanal	78-84-2	C4H8O	72.1	835.1	174.022	1.27829	0.0680±0.0060	0.0519±0.0105	0.0314±0.0012	1.1345±0.0054	1.1971±0.0172	banana, melon , slightly nutty
3-Methyl butanal	590-86-3	C5H10O	86.1	936.5	214.927	1.40838	0.0237±0.0010	0.0537±0.0117	0.0044±0.0004	0.3424±0.0079	0.3326±0.0144	chocolate, fat
Propanal-M	123-38-6	C3H6O	58.1	819.6	168.819	1.07706	0.0153±0.0009	0.1171±0.0211	0.0112±0.0008	0.0907±0.0067	0.0943±0.0042	pungent, green grassy
(E)-2-Heptenal	18829-55-5	C7H12O	112.2	1323.9	667.955	1.2538	0.0094±0.0007	0.0082±0.0004	0.0030±0.0002	0.0669±0.0155	0.0752±0.0030	spicy, green vegetables, fresh, fatty

1-nonanal	124-19-6	C9H18O	142.2	1401.3	782.658	1.4784	n.d.	n.d.	n.d.	0.0319±0.0025	0.0432±0.0017	rose, citrus, strong oily
(Z)-2-heptenal	57266-86-1	C7H12O	112.2	1311.4	651.037	1.2424	0.0059±0.0003	0.0425±0.0072	n.d.	0.0249±0.0024	0.0247±0.0018	null
(Z)-2-pentenal-D	1576-86-9	C5H8O	84.1	1108.6	351.211	1.35002	n.d.	0.0168±0.0037	n.d.	n.d.	n.d.	null
(Z)-2-pentenal-M	1576-86-9	C5H8O	84.1	1109.6	352.431	1.09137	n.d.	0.0904±0.0148	n.d.	n.d.	n.d.	null
Propanal-D	123-38-6	C3H6O	58.1	818.8	168.55	1.14001	n.d.	0.0857±0.0189	n.d.	n.d.	n.d.	pungent, green grassy
**Ketones**												
2-propanone	67-64-1	C3H6O	58.1	843.2	176.778	1.11602	0.1402±0.0054	0.3465±0.0528	0.0771±0.0035	0.4698±0.0164	0.5036±0.0042	fresh, apple, pear
2-hydroxy-2-methyl-4-pentanone-M	123-42-2	C6H12O2	116.2	1365.3	726.993	1.13982	0.4627±0.0064	0.3082±0.0326	0.2774±0.0179	1.6478±0.1255	2.3256±0.0246	Mild, pleasant
2-Pentanone	107-87-9	C5H10O	86.1	995.7	251.268	1.37321	0.3651±0.0189	0.9685±0.1246	0.2301±0.0231	1.8841±0.1823	1.8989±0.0765	acetone, fresh, sweet fruity, wine
2-hydroxy-2-methyl-4-pentanone-D	123-42-2	C6H12O2	116.2	1362.3	722.631	1.53098	2.0313±0.0834	0.2488±0.0384	1.3697±0.0531	0.8649±0.1818	2.5033±0.1188	Mild, pleasant
2,3-pentadione	600-14-6	C5H8O2	100.1	1070.4	311.444	1.20486	0.0917±0.0029	0.0571±0.0090	0.0497±0.0016	0.4587±0.0165	0.4564±0.0046	sweet, cream, caramel, nuts, cheese
2-Heptanone-D	110-43-0	C7H14O	114.2	1187	456.212	1.62432	0.04260±0.0020	0.2779±0.0356	0.0222±0.0045	0.2775±0.0319	0.3072±0.0145	pear, banana, fruity, slight medicinal fragrance
2-Heptanone-M	110-43-0	C7H14O	114.2	1188.9	458.806	1.2619	0.1306±0.0034	0.2129±0.0253	0.0679±0.0074	0.6605±0.0197	0.6115±0.0149	pear, banana, fruity, slight medicinal fragrance
2-Butanone, 3-hydroxy	513-86-0	C4H8O2	88.1	1299.5	635.397	1.06616	0.0085±0.0002	0.0242±0.0054	0.0076±0.0005	n.d.	n.d.	butter, cream
2-Hexanone	591-78-6	C6H12O	100.2	1093.1	333.327	1.49306	0.1022±0.0043	0.5305±0.0558	0.0718±0.0165	0.5633±0.1060	0.5620±0.0357	fruity, fungal, meaty, buttery
2-Butanone	78-93-3	C4H8O	72.1	927.7	210.05	1.23536	0.0156±0.0001	0.4707±0.0637	0.0108±0.0037	0.0871±0.0230	0.0954±0.0125	fruity , camphor
**Furan derivatives**												
2-furaldehyde	98-01-1	C5H4O2	96.1	1471.9	904.29	1.08864	n.d.	n.d.	n.d.	0.0433±0.0007	0.0805±0.0044	sweet, woody, almond, bready
**Other categories**												
2	unidentified	*	0	960.4	228.694	1.41966	0.0155±0.0010	0.0196±0.0025	0.0386±0.0021	0.1865±0.0029	0.1598±0.0028	null
3	unidentified	*	0	1197.5	470.807	1.78918	0.2487±0.0088	0.4148±0.0561	0.0554±0.0023	1.6661±0.0992	1.6563±0.0205	null
4	unidentified	*	0	1562.1	1087.543	1.59351	0.0313±0.0022	0.1281±0.0270	n.d.	0.1531±0.0244	0.1509±0.0134	null
5	unidentified	*	0	1308.3	647.022	0.94927	0.0800±0.0025	0.0930±0.0095	0.0670±0.0019	0.7243±0.0216	0.7648±0.0157	null
6	unidentified	*	0	1000	254.431	1.59128	0.0220±0.0015	0.0193±0.0041	0.0061±0.0002	0.0689±0.0019	0.0661±0.0015	null
7	unidentified	*	0	1670.8	1358.208	1.84283	n.d.	0.3072±0.0686	n.d.	n.d.	n.d.	null
8	unidentified	*	0	1408.4	794.021	1.5072	n.d.	0.0318±0.0045	n.d.	n.d.	n.d.	null
9	unidentified	*	0	1340.4	690.848	1.34629	0.1719±0.0032	0.1898±0.0202	0.0726±0.0026	0.7909±0.0089	0.7603±0.0088	null
1	unidentified	*	0	1291.9	624.587	1.23857	0.0726±0.0032	0.0410±0.0062	0.0517±0.0003	0.1079±0.0064	0.1580±0.0067	null

Annotation: The mean ± SD of measurements made in triplicates were used to reflect the characteristic values, and 3, 2-methyl-3-heptanone used as internal standards. The suffixes M, D or T represent the monomer, dimer and trimer of the same substance respectively; "n.d." stands for "not detected"; the described odors are mainly based on databases, such as https://www.flavornet.org and https://www.femaflavor.org.

Abbreviations: HS-GC-IMS, headspace-gas chromatography-ion mobility spectrometry; CAS, chemical abstracts service number; Dt [a.u.]: drift time; MW, molecular weight; RI, retention index; Rt [sec]: retention time; VOC, volatile organic compound; JD, special grades of NXB, 77.0 ± 0.1% ethanol by volume; YZ, superior grades of NXB, 72.0 ± 0.1% ethanol by volume; NXB, Nongxiangxing Baijiu; HS, Huangshui; FG, Fermented grains; PM, Pit mud.
