# Supplementary material for: Comparative Volatile Profiling Reveals the Chemical Association of Huangshui with Fermented Grains, Pit Mud, and Nongxiangxing Baijiu
Source: Foods. 2026 Aug 2;15(15):2722. doi: 10.3390/foods15152722 (PMC13463836; doi:10.3390/foods15152722)
Supplement: Supplementary file 1 [file foods-15-02722-s001.zip › Table-S3.pdf]

Table S3. The relative contents of VOCs detected in 5 different samples via HS-SPME-GC-MS.

**Compounds**	**CAS**	**Lib-RI**	**HS-RI**	**PM-RI**	**FG-RI**	**JD / YZ-RI**	**FG** / (mg·Kg^{-1})	**HS** / (mg·L^{-1})	**PM** / (mg·Kg^{-1})	**JD** / (mg·L^{-1})	**YZ** / (mg·L^{-1})
**Alcohols**											
1-Butanol	71-36-3	659	1163	/	1262	1090	0.122±0.010	n.d.	0.937±0.148	52.133±5.950	33.134±2.952
1-Pentanol	71-41-0	765	1219	/	/	/	0.457±0.027	n.d.	n.d.	n.d.	n.d.
2-Heptanol	543-49-7	900	1325	/	/	/	0.014±0.001	n.d.	n.d.	n.d.	n.d.
1-Hexanol	111-27-3	868	1360	1344	1514	1298	0.588±0.036	5.683±0.238	6.199±1.178	n.d.	162.008±40.407
1-Heptanol	111-70-6	970	1461	/	/	/	0.043±0.003	n.d.	n.d.	n.d.	n.d.
1-Octanol	111-87-5	1070	1562	1550	1791	/	0.134±0.004	12.572±3.646	0.320±0.013	n.d.	n.d.
2-Methylpropanol	78-83-1	624	/	/	1209	1028	n.d.	n.d.	0.050±0.040	20.060±1.264	12.110±3.431
3-Methylbutanol	123-51-3	736	/	/	1334	1157	n.d.	n.d.	2.258±0.396	218.817±52.822	136.235±32.573
2-Butanol	78-92-2	598	/	/	/	947	n.d.	n.d.	n.d.	3.636±1.281	n.d.
1-Nonanol	143-08-8	1173	/	/	/	1594	n.d.	n.d.	n.d.	n.d.	3.262±0.537
**Acids**											
Propanoic acid	79-09-4	706	1558	1524	1761	/	0.072±0.001	1.351±0.341	0.417±0.140	n.d.	n.d.
2-MethylPropanoic acid	79-31-2	765	1586	1553	1799	/	0.044±0.001	1.810±0.170	0.455±0.176	n.d.	n.d.
Butanoic acid	107-92-6	802	1640	1613	1878	1560	1.725±0.028	36.403±13.955	7.545±4.206	4.997±4.328	7.819±0.113
Pentanoic acid	109-52-4	901	1759	1729	2034	1669	1.501±0.091	23.225±2.039	4.240±1.671	2.596±0.324	6.004±1.167
4-Methylpentanoic acid	646-07-1	949	1821	1795	2128	/	0.064±0.003	1.195±0.239	0.642±0.069	n.d.	n.d.
Heptenoic acid	111-14-8	1080	1971	/	2339	1884	2.064±0.121	n.d.	10.129±4.058	5.174±0.230	13.003±4.757
Octanoic acid	124-07-2	1180	2079	/	2480	1990	3.108±0.087	n.d.	6.110±0.878	6.974±1.168	17.542±5.986
Nonanoic acid	112-05-0	1273	2176	2161	2612	/	0.119±0.015	4.654±0.256	0.144±0.037	n.d.	n.d.
n-Decanoic acid	334-48-5	1372	2265	2268	2742	/	0.141±0.028	3.619±1.182	0.131±0.114	n.d.	n.d.
Benzoic acid	65-85-0	1177	2399	2418	2937	/	0.248±0.061	0.373±0.044	0.358±0.124	n.d.	n.d.
Dodecanoic acid	143-07-7	1567	2425	/	/	/	0.191±0.082	n.d.	n.d.	n.d.	n.d.
Hydrocinnamic acid	501-52-0	1356	2552	2582	3198	/	0.082±0.020	0.181±0.003	0.176±0.008	n.d.	n.d.
Tetradecanoic acid	544-63-8	1768	2605	/	/	/	0.168±0.024	n.d.	n.d.	n.d.	n.d.
Acetic acid	64-19-7	610	/	/	1636	1395	n.d.	n.d.	3.311±0.496	3.808±0.317	2.613±0.207
3-Methylbutanoic acid	503-74-2	850	/	/	1939	1600	n.d.	n.d.	2.196±1.044	2.195±0.163	3.857±1.479
Hexanoic acid	142-62-1	990	/	/	/	1777	n.d.	n.d.	n.d.	n.d.	0.496±0.064
**Esters**											
Ethyl butyrate	105-54-4	802	1043	/	1144	961	2.868±0.072		0.972±0.047	868.778±194.948	459.666±56.805
Ethyl 2-methylbutyrate	7452-79-1	849	1055	/	/	979	0.034±0.010		n.d.	11.420±2.858	4.967±0.542
Ethyl 3-methylbutyrate	108-64-5	853	1067	/	/	999	0.180±0.002		n.d.	22.554±3.604	9.111±2.640
Ethyl pentanoate	539-82-2	900	1125	1128	1253	1081	2.946±0.280	0.157±0.021	2.463±0.026	1845.310±375.109	981.552±106.609
Ethyl 4-methylpentanoate	25415-67-2	969	1181	/	/	1137	0.096±0.017	n.d.	n.d.	n.d.	n.d.
Butyl butyrate	109-21-7	995	1210	/	/	/	0.102±0.002	n.d.	n.d.	n.d.	n.d.
Isobutyl isovalerate	589-59-3	1005	1252	/	1394	1217	0.010±0.002	n.d.	0.017±0.000	0.787±0.147	n.d.
Isoamyl butyrate	106-27-4	1056	1261	/	1405	1225	0.093±0.011	n.d.	0.220±0.003	66.009±15.237	32.442±9.183
Hexyl acetate	142-92-7	1011	1270	1259	1413	1230	0.259±0.022	0.051±0.034	0.382±0.220	135.463±30.846	67.593±18.333
Ethyl 5-methylhexanoate	10236-10-9	1068	1283	1272	1430	1240	0.099±0.018	0.257±0.007	0.177±0.066	52.949±3.679	30.705±4.498
Butyl pentanoate	591-68-4	1093	1310	/	/	1263	0.087±0.002	n.d.	n.d.	67.461±15.582	35.329±10.097
Pentanoate	2445-72-9	1057	1312	/	/	/	0.018±0.002	n.d.	n.d.	n.d.	n.d.
Propyl hexanoate	626-77-7	1094	1314	1304	1470	1268	1.026±0.127	2.479±0.738	2.350±0.725	587.919±141.119	301.033±87.081

Ethyl heptanoate	106-30-9	1098	1332	1327	1493	1285	6.001±0.008	30.800±1.629	9.338±1.936	2246.311±133.361	1250.530±326.930
2-Methylpropyl hexanoate	105-79-3	1149	1348	1337	/	1298	0.246±0.050	2.195±1.414	n.d.	315.679±22.791	7.057±2.052
(L)-2-Hydroxyethyl propionate	687-47-8	848	1355	1334	/	1288	0.777±0.034	1.164±0.101	n.d.	37.760±8.726	57.587±14.656
Butyl hexanoate	626-82-4	1189	1409	1612	1594	1359	1.602±0.119	5.016±0.587	4.459±0.090	1158.520±48.819	709.513±191.101
Hexyl butyrate	2639-63-6	1192	1412	/	/	1360	0.325±0.024	n.d.	n.d.	121.118±3.56	73.203±19.613
Ethyl octanoate	106-32-1	1196	1435	1426	1628	1383	9.041±0.074	75.595±10.415	11.261±0.355	2595.120±150.176	1487.731±434.860
Isoamyl hexanoate	2198-61-0	1250	1455	1452	1658	1404	1.295±0.196	37.495±0.463	6.380±0.103	1008.051±45.428	560.206±150.022
Methyl formate	107-31-3	372	1466	/	/	/	1.345±0.290	n.d.	n.d.	n.d.	n.d.
Pentyl hexanoate	540-07-8	1287	1507	1495	1732	1450	0.308±0.004	1.091±0.108	1.812±0.216	358.601±120.545	151.948±31.267
Propyl octanoate	624-13-5	1290	1516	/	/	1456	0.035±0.010	n.d.	n.d.	19.368±0.246	11.832±0.904
Butyl lactate	138-22-7	1047	1528	/	1742	/	0.018±0.001	n.d.	0.336±0.009	n.d.	n.d.
Ethyl pelargonate	123-29-5	1295	1533	1521	/	1471	0.232±0.040	0.633±0.233	n.d.	96.294±0.231	60.883±3.046
Ethyl 2-hydroxy-4-methylpentanoate	10348-47-7	1060	1549	/	1771	1477	0.229±0.003	n.d.	1.355±0.162	11.532±0.589	26.125±0.768
Hexyl hexanoate	6378-65-0	1384	1605	1597	1866	1548	2.328±0.375	8.730±1.835	7.169±6.262	1781.171±156.095	1304.167±9.897
Ethyl decanoate	110-38-3	1396	1633	1647	1923	1572	0.545±0.044	24.618±8.721	0.64±0.032	254.574±17.463	208.121±1.405
3-Methylbutyl octanoate	2035-99-6	1446	1652	1666	/	1590	0.027±0.004	9.084±1.733	n.d.	25.966±6.848	16.886±0.806
Diethyl succinate	123-25-1	1181	1678	1670	1958	1610	0.453±0.025	0.669±0.163	1.656±0.339	3.183±0.614	8.636±0.847
Hexyl octanoate	1117-55-1	1581	1812	/	/	1743	0.280±0.088	n.d.	n.d.	20.629±1.538	21.322±0.434
Ethyl dodecanoate	106-33-2	1594	1855	1854	/	1777	0.177±0.013	3.341±1.036	n.d.	146.933±17.434	57.045±15.623
2,2,4-Trimethylpentanediol isobutyl ester	6846-50-0	1588	1876	/	/	/	0.023±0.004	n.d.	n.d.	n.d.	n.d.
Methyl hexanoate	106-70-7	925	1914	/	2276	/	0.103±0.018	n.d.	0.697±0.329	n.d.	n.d.
Propan-2-yl nonanone	104-61-0	1365	2039	2031	2460	/	0.046±0.003	0.263±0.049	0.247±0.111	n.d.	n.d.
Ethyl tetradecanoate	124-06-1	1793	2050	/	2473	1981	0.692±0.066	n.d.	0.707±0.062	89.085±9.425	105.118±26.010
Ethyl pentadecanoate	41114-00-5	1894	2147	2115	2597	2079	0.130±0.021	0.494±0.028	0.074±0.011	4.860±0.170	6.384±0.004
Ethyl hexadecanoate	628-97-7	1993	2238	2253	2727	2175	4.736±0.181	3.743±0.902	4.199±0.160	389.534±50.485	376.364±28.123
Ethyl 9-hexadecenoate	54546-22-4	1976	2259	/	2756	2195	0.314±0.016	n.d.	0.197±0.033	12.921±2.029	13.347±5.821
Succinic acid monoethyl ester	1070-34-4	1141	2358	/	2873	/	0.074±0.002	n.d.	0.238±0.029	n.d.	n.d.
Ethyl octadecanoate	111-61-5	2195	2395	2436	2964	2349	0.064±0.008	0.101±0.022	0.030±0.004	2.331±0.484	2.644±0.104
Ethyl oleate	111-62-6	2168	2411	2453	2990	2365	0.948±0.142	0.590±0.179	0.669±0.230	44.353±12.016	47.221±4.747
Ethyl (E)-octadec-9-enoate	6114-18-7	2174	2417	/	/	/	0.080±0.013	n.d.	n.d.	n.d.	n.d.
Ethyl linoleate	544-35-4	2160	2448	2492	3053	2403	0.551±0.161	0.081±0.044	0.365±0.054	13.853±4.729	18.168±0.432
butyl acetate	123-86-4	812	/	/	1177	1004	n.d.	n.d.	0.352±0.006	20.197±0.372	12.724±0.379
Propyl 2-hydroxypropionate	616-09-1	947	/	/	1611	/	n.d.	n.d.	0.062±0.012	n.d.	n.d.
Heptyl formate	112-23-2	1039	/	/	1650	/	n.d.	n.d.	0.260±0.161	n.d.	n.d.
Ethyl nicotinate	614-18-6	1218	/	/	2212	/	n.d.	n.d.	0.017±0.005	n.d.	n.d.
Diethyl suberate	2050-23-9	1583	/	/	2546	/	n.d.	n.d.	0.009±0.000	n.d.	n.d.
Ethyl cinnamate	103-36-6	1464	/	/	2588	2062	n.d.	n.d.	0.006±0.002	0.362±0.094	0.329±0.069
Diethyl azelate	624-17-9	1688	/	/	2677	/	n.d.	n.d.	0.038±0.002	n.d.	n.d.
Ethyl acetate	141-78-6	612	/	/	/	745	n.d.	n.d.	n.d.	998.510±24.455	509.531±130.205
Ethyl propionate	105-37-3	710	/	/	/	864	n.d.	n.d.	n.d.	0.271±0.135	6.198±2.664
Ethyl 2-methylpropionate	97-62-1	756	/	/	/	876	n.d.	n.d.	n.d.	15.077±0.826	2.938±0.305
n-Propyl acetate	109-60-4	712	/	/	/	894	n.d.	n.d.	n.d.	9.559±2.003	n.d.
Isobutyl acetate	110-19-0	772	/	/	/	933	n.d.	n.d.	n.d.	9.116±1.166	2.486±0.112
Isopentyl acetate	123-92-2	876	/	/	/	1061	n.d.	n.d.	n.d.	21.678±1.668	63.253±2.262

2-Methylpropyl pentanoate	10588-10-0	1044	/	/	/	1219	n.d.	n.d.	n.d.	26.775±3.550	3.498±1.047
Ethyl 3-hexenoate	2396-83-0	1005	/	/	/	1255	n.d.	n.d.	n.d.	0.740±0.056	2.305±0.096
Hexyl propionate	2445-76-3	1108	/	/	/	1287	n.d.	n.d.	n.d.	4.548±0.735	2.233±0.323
Propyl caprylate	7778-87-2	1183	/	/	/	1362	n.d.	n.d.	n.d.	7.093±0.413	10.159±2.403
Hexyl 3-methylbutyrate	10032-13-0	1244	/	/	/	1388	n.d.	n.d.	n.d.	18.332±1.153	n.d.
Isobutyl enanthate	7779-80-8	1218	/	/	/	1395	n.d.	n.d.	n.d.	1.744±0.029	n.d.
Octyl acetate	112-14-1	1210	/	/	/	1416	n.d.	n.d.	n.d.	8.520±1.044	2.056±0.545
Heptyl isobutyrate	2349-13-5	1247	/	/	/	1453	n.d.	n.d.	n.d.	0.344±0.039	1.596±0.073
Heptyl butyrate	5870-93-9	1296	/	/	/	1453	n.d.	n.d.	n.d.	3.163±0.484	n.d.
Octanoic acid isobutyl ester	5461-06-3	1348	/	/	/	1486	n.d.	n.d.	n.d.	0.332±0.027	4.013±1.276
3-Methylbutyl caprylate	109-25-1	1347	/	/	/	1491	n.d.	n.d.	n.d.	6.721±1.323	19.378±0.425
2-Methyl octyl propionate	109-15-9	1344	/	/	/	1551	n.d.	n.d.	n.d.	7.441±0.438	1.674±0.407
Ethyl trans-4-decenoate	76649-16-6	1347	/	/	/	1615	n.d.	n.d.	n.d.	3.540±1.499	3.264±0.293
Dibutyl 2-ethylmalonate	6976-72-3	1482	/	/	/	1643	n.d.	n.d.	n.d.	3.293±0.128	45.480±0.354
Ethyl undecanoate	627-90-7	1494	/	1756	/	1673	n.d.	1.194±0.201	n.d.	14.009±2.601	5.843±0.940
Ethyl trans-2-decenoate	7367-88-6	1389	/	/	/	1692	n.d.	n.d.	n.d.	7.942±3.775	4.593±0.576
Ethyl 3-nonenoate	91213-30-8	1290	/	/	/	1517	n.d.	n.d.	n.d.	n.d.	0.992±0.310
Methyl salicylate	119-36-8	1192	/	/	/	1709	n.d.	n.d.	n.d.	n.d.	1.035±0.372
Ethyl hexanoate	123-66-0	999	/	1223	/	/	n.d.	88.819±5.235	71.503±2.001	11596.213±2890.644	9912.332±743.750
Ethyl tridecanoate	28267-29-0	1687	/	1901	/	/	n.d.	1.491±0.391	n.d.	n.d.	n.d.
Octyl heptanoate	5132-75-2	1680	/	1917	/	/	n.d.	5.753±0.005	n.d.	n.d.	n.d.
**Aldehydes and ketones**											
Nonanal	124-19-6	1104	1390	/	1572	1336	0.120±0.012	n.d.	0.063±0.019	8.539±0.109	10.366±0.032
N-methyl-2-pyrrolecarboxaldehyde	1192-58-1	1016	2127	/	/	/	0.004±0.000	n.d.	n.d.	n.d.	n.d.
2-Methylpropanal	78-84-2	552	/	/	/	622	n.d.	n.d.	n.d.	0.340±0.064	1.174±0.234
3-Methylbutanal	590-86-3	652	/	/	/	808	n.d.	n.d.	n.d.	1105.988±247.342	21.426±4.806
(E)-2-Octenal	2548-87-0	1060	/	/	/	1372	n.d.	n.d.	n.d.	n.d.	2.592±0.917
2-Pentadecanone	2345-28-0	1698	2021	/	/	/	0.027±0.001	n.d.	n.d.	n.d.	n.d.
2-Nonanone	821-55-6	1092	/	/	/	1331	n.d.	n.d.	n.d.	3.397±0.344	4.679±1.275
2-Undecanone	112-12-9	1294	/	/	/	1530	n.d.	n.d.	n.d.	1.183±0.165	6.888±0.293
(E)-6,10-dimethylundeca-5,9-dien-2-one	3796-70-1	1453	/	/	/	1787		n.d.	n.d.	n.d.	264.268±15.327
**Acetals**											
1,1-Diethoxy-3-methylbutane	3842-03-3	955	/	/	/	1010	n.d.	n.d.	n.d.	3.941±1.139	10.252±0.170
1,1-Diethoxynonane	54815-13-3	1381	/	/	/	1466	n.d.	n.d.	n.d.	2.562±0.281	20.855±3.473
**Aromatic and phenolic compounds**											
Styrene	100-42-5	893	1255	1243	1402	1222	0.013±0.001	0.447±0.018	0.157±0.010	2.083±0.375	1.318±0.189
Benzaldehyde	100-52-7	962	1524	1510	1759	1462	0.378±0.009	1.087±0.195	0.683±0.410	14.896±1.163	14.940±0.296
Benzeneacetaldehyde	122-78-1	1045	1642	/	/	/	0.064±0.001	n.d.	n.d.	n.d.	n.d.
Acetophenone	98-86-2	1066	1646	/	/	/	0.011±0.001	n.d.	n.d.	n.d.	n.d.
Ethyl benzoate	93-89-0	1172	1661	/	1951	1597	0.049±0.005	n.d.	0.064±0.020	13.872±0.357	18.885±0.621
Naphthalene	91-20-3	1182	1738	/	2063	1671	0.084±0.005	n.d.	0.029±0.005	2.894±0.727	2.426±0.108
3,4-Dimethylbenzaldehyde	5973-71-7	1236	1739	/	2171	/	0.009±0.002	n.d.	0.109±0.017	n.d.	n.d.
Ethyl phenylacetate	101-97-3	1247	1790	/	/	1718	2.240±0.193	n.d.	n.d.	220.306±44.873	312.399±5.192
Propyl phenylacetate	4606-15-9	1331	1875	/	2237	/	0.005±0.000	n.d.	0.034±0.007	n.d.	n.d.

Ethyl phenylpropionate	2021-28-5	1353	1889	1884	2261	1816	1.241±0.008	6.758±1.280	2.989±0.301	124.116±24.359	256.485±24.375
Phenethyl alcohol	60-12-8	1116	1923	1910	2296	1847	1.364±0.118	1.351±0.175	1.922±0.750	3.040±0.429	5.440±0.465
Butyl phenylacetate	122-43-0	1440	1965	1963	/	1891	0.016±0.000	0.142±0.029	n.d.	n.d.	3.084±1.253
2-Methoxy-5-methylphenol	1195-09-1	1193	1969	1957	2356	/	0.178±0.010	0.519±0.086	0.642±0.321	n.d.	n.d.
Methyl 2-pyrrolyl ketone	1072-83-9	1063	1988	/	2381	/	0.009±0.000	n.d.	0.014±0.008	n.d.	n.d.
Phenol	108-95-2	981	2029	2002	2416	1942	0.323±0.031	3.496±0.517	1.248±0.489	0.836±0.128	0.914±0.017
4-Ethyl-2-methoxyphenol	2785-89-9	1282	2045	2030	2452	1964	0.117±0.027	0.446±0.023	0.445±0.210	n.d.	0.670±0.175
p-Cresol	106-44-5	1077	2104	/	/	2017	2.694±0.340	n.d.	n.d.	8.209±1.466	10.318±3.411
4-Ethylphenol	123-07-9	1169	2189	2176	2633	2103	0.125±0.024	0.650±0.114	0.419±0.186	0.500±0.069	0.861±0.326
2-Hydroxy-3-phenylpropionic acid ethyl ester	15399-05-0	1432	2272	2280	2763	2192	0.603±0.171	0.449±0.013	0.837±0.309	1.019±0.263	1.226±0.147
2,4-Di-tert-butylphenol	96-76-4	1514	2296	/	2790	2225	1.014±0.171	n.d.	0.067±0.008	5.535±0.903	4.583±1.272
3-Methylindole	83-34-1	1391	2443	/	/	/	0.029±0.004	n.d.	n.d.	n.d.	n.d.
Phenylacetic acid	103-82-2	1262	2496	2522	3097	1798	0.665±0.219	0.489±0.054	1.498±0.410	n.d.	1.323±0.102
4-Vinylbenzoic acid	1075-49-6	1353	2695	/	/	/	0.011±0.001	n.d.	n.d.	n.d.	n.d.
Phenethyl acetate	103-45-7	1258	/	/	2158	1748	n.d.	n.d.	0.363±0.150	9.058±1.836	19.392±1.282
1,2-Ethylene glycol monobenzoate	94-33-7	1402	/	/	2238	/	n.d.	n.d.	0.008±0.000	n.d.	n.d.
Butyl phenethyl ester	103-52-6	1444	/	/	2366	1894	n.d.	n.d.	0.053±0.010	1.630±0.082	3.878±1.567
3-MethylPhenol	108-39-4	1075	/	2082	2516	/	n.d.	26.909±0.212	5.711±1.879	n.d.	n.d.
2-Phenylethyl hexanoate	6290-37-5	1650	/	2170	2628	2093	n.d.	3.438±0.200	0.269±0.020	9.248±0.524	15.502±2.978
2-Methoxy-4-vinylphenol	7786-61-0	1316	/	/	2668	2125	n.d.	n.d.	0.017±0.000	n.d.	n.d.
2-Methylindole	95-20-5	1398	/	2473	3024	/	n.d.	1.925±0.343	0.134±0.027	n.d.	n.d.
4-Pentylphenol	14938-35-3	1464	/	/	/	1424	n.d.	n.d.	n.d.	4.711±0.731	n.d.
(2,2-Diethoxyethyl) benzene	6314-97-2	1328	/	/	/	1649	n.d.	n.d.	n.d.	58.906±6.755	24.394±6.454
2-Methylnaphthalene	91-57-6	1297	/	/	/	1786	n.d.	n.d.	n.d.	4.620±1.311	0.914±0.123
Isobutyl phenylacetate	102-13-6	1393	/	1888	/	1891	n.d.	0.026±0.006	n.d.	1.382±0.373	n.d.
Isopropyl 3-phenylpropionate	22767-95-9	1385	/	/	/	1903	n.d.	n.d.	n.d.	1.552±0.308	0.862±0.337
2-Ethenylnaphthalene	827-54-3	1407	/	/	/	1920	n.d.	n.d.	n.d.	0.430±0.090	0.665±0.039
Biphenyl	92-52-4	1381	/	/	/	1920	n.d.	n.d.	n.d.	0.766±0.227	n.d.
1,6-Dimethyl-4- (1-methylethyl) naphthalene	483-78-3	1674	/	/	/	2140	n.d.	n.d.	n.d.	0.649±0.079	n.d.
4-Methoxystyrene	637-69-4	1156	/	/	/	1612	n.d.	n.d.	n.d.	n.d.	6.439±2.668
Acenaphthene	83-32-9	1482	/	/	/	1920	n.d.	n.d.	n.d.	n.d.	1.895±0.202
Phenethyl 2-methylbutyrate	24817-51-4	1488	/	/	/	1995	n.d.	n.d.	n.d.	n.d.	0.636±0.028
3-Phenylthiophene	2404-87-7	1368	/	/	/	2063	n.d.	n.d.	n.d.	n.d.	1.658±0.627
1,3-Benzenediolmonobenzoate	136-36-7	1855	/	1276	/	/	n.d.	0.066±0.006	n.d.	n.d.	n.d.
2-Phenylbutanal	4411-89-6	1276	/	1932	/	/	n.d.	0.053±0.010	n.d.	n.d.	n.d.
3-Phenylpropanol	122-97-4	1233	/	2047	/	/	n.d.	0.046±0.000	n.d.	n.d.	n.d.
3-Ethylphenol	620-17-7	1169	/	2183	/	/	n.d.	0.325±0.056	n.d.	n.d.	n.d.
3-Ethylindole	1484-19-1	1482	/	2548	/	/	n.d.	0.055±0.006	n.d.	n.d.	n.d.
**Terpenes**											
α-Terpenene	99-86-5	1017	1273	/	/	/	0.004±0.000	n.d.	n.d.	n.d.	n.d.
α-Curcumene	644-30-4	1483	/	1781	/	/	n.d.	2.488±0.175	n.d.	n.d.	n.d.
**Furans**											
Furfural	98-01-1	833	1473	/	/	1410	0.029±0.001	n.d.	n.d.	7.898±1.866	12.534±3.002

1-(2-Furanyl)-1-Propanone	3194-15-8	1012	1579	/	/	/	0.005±0.000	n.d.	n.d.	n.d.	n.d.
2-Furanmethanol	98-00-0	860	1670	/	/	/	0.085±0.000	n.d.	n.d.	n.d.	n.d.
Furfuryl hexanoate	39252-02-3	1368	1874	1868	2230	1800	0.024±0.001	0.611±0.151	0.050±0.008	10.377±0.071	14.644±0.154
2,5-Dimethylfuran	625-86-5	707	/	/	/	857	n.d.	n.d.	n.d.	36.509±3.553	n.d.
Furfuryl acetate	623-17-6	995	/	/	/	1474	n.d.	n.d.	n.d.	12.383±0.991	0.428±0.038
2-Methylbenzofuran	4265-25-2	1109	/	/	/	1525	n.d.	n.d.	n.d.	34.587±1.608	n.d.
2,2'-Bifuran	5905-00-0	1047	/	/	/	1529	n.d.	n.d.	n.d.	0.775±0.131	0.661±0.203
3-Phenylfuran	13679-41-9	1226	/	/	/	1789	n.d.	n.d.	n.d.	1.072±0.223	1.042±0.010
**Sulfur compounds**											
Dimethyl trisulfide	3658-80-8	971	1374	/	/	1316	0.003±0.000	n.d.	n.d.	n.d.	3.223±1.702
Ethyl 3-(methylthio)propionate	13327-56-5	1098	1569	/	1807	1497	0.023±0.000	n.d.	0.066±0.037	1.176±0.344	2.659±0.020
3-Methylthiopropanol	505-10-2	981	1728	/	/	/	0.018±0.004	n.d.	n.d.	n.d.	n.d.
Methanethiol	74-93-1	401	/	/	/	516	n.d.	n.d.	n.d.	n.d.	0.131±0.005
S-Methyl pentanethioate	42075-43-4	942	/	/	/	1248	n.d.	n.d.	n.d.	6.944±1.841	n.d.
S-Methyl thiobutyrate	2432-51-1	888	/	/	/	1143	n.d.	n.d.	n.d.	0.237±0.015	n.d.
**Other compounds**											
Dodecane	112-40-3	1200	1171	/	/	/	0.020±0.006	n.d.	n.d.	n.d.	n.d.
Heptadecane	629-78-7	1700	1693	/	/	/	0.017±0.001	n.d.	n.d.	n.d.	n.d.
Hexanoic anhydride	2051-49-2	1517	1953	/	2329	/	0.032±0.007	n.d.	0.264±0.096	n.d.	n.d.
Propionic anhydride	123-62-6	921	/	/	/	1157	n.d.	n.d.	n.d.	94.772±5.617	n.d.

Annotation: The mean ± SD of measurements made in triplicates were used to reflect the characteristic values."n.d." stands for "not detected"; "/" stands for "no detected RI".

Abbreviations: HS-SPME-GC-MS, headspace solid-Phase microextraction-gas chromatography-mass spectrometry; CAS, chemical abstracts service number; VOC, volatile organic compound; RI, retention index; Lib-RI, retention index searched in the NIST12 mass spectral library; JD, special grades of NXB, 77.0 ± 0.1% ethanol by volume; YZ, superior grades of NXB, 72.0 ± 0.1% ethanol by volume; NXD, Nongxiangxing Baijiu; HS, Huangshui; FG, Fermented grains; PM, Pit mud.
